# Advancing Health Equity Through Telepharmacy Antimicrobial Stewardship in Rural Communities in the United States

**DOI:** 10.7759/cureus.100020

**Published:** 2025-12-24

**Authors:** Adeola Bakare, Patrick Oliorah, Daniel O Umoru, Chidinma V Muoghalu, Mary-Jane E Ugbor, Rebecca Omachonu

**Affiliations:** 1 Pharmacy, Citiserve Pharmacy Ltd, Lagos, NGA; 2 Pharmaceutical Economics and Policy, Chapman University, Irvine, USA; 3 College of Pharmaceutical Sciences, University of Nigeria Nsukka, Enugu, NGA; 4 Pharmacy, National Hospital Abuja, Abuja, NGA; 5 Pharmacy, Chapman University, Irvine, USA; 6 Biochemistry and Nutrition, Nigeria Institute of Medical Research, Lagos, NGA; 7 Pharmaceutical Sciences, University of PortHarcourt, PortHarcourt, NGA; 8 Pharmacy, Keck Graduate Institute of Applied Life Sciences, Claremont, USA

**Keywords:** antimicrobial resistance, antimicrobial stewardship, digital health, health equity, health policy, pharmacy, public health policy interdisciplinary relevance: public health, rural healthcare, sustainable development goals, telepharmacy

## Abstract

Antimicrobial resistance remains a growing global threat, disproportionately impacting underserved rural communities in the United States with limited access to pharmacy services. This meta review synthesizes evidence on community-led telepharmacy antimicrobial stewardship programs and evaluates their impact on antibiotic prescribing practices, resistance patterns, and alignment with Sustainable Development Goals. The objective is to determine the clinical, economic, and equity outcomes associated with telepharmacy-supported stewardship in rural United States healthcare settings.

From 8,742 identified records, 20 studies met the inclusion criteria spanning telepharmacy implementation, antimicrobial stewardship outcomes, prescribing patterns, and policy frameworks. Studies published between 2005 and 2021 provided quantifiable metrics on clinical, economic, and equity-related outcomes.

Inappropriate antibiotic use decreased by 28.6% (95% CI 21.3 to 35.9, p<0.001), and odds of appropriate prescribing improved significantly (OR 3.21, 95% CI 2.54 to 4.06). Broad spectrum utilization declined by 32.4%, guideline concordant selection improved by 41.2%, medication errors decreased by 94% (OR 0.06, 95 percent CI 0.03 to 0.12), and antibiotic de-escalation rates nearly doubled (34% to 67%).

Hospitals integrating telepharmacy stewardship experienced reduced length of stay (mean 1.8 days, 95% CI 1.2 to 2.4, p<0.001) and 41% fewer Clostridium difficile infections (IRR 0.59, 95 percent CI 0.43 to 0.81). Days of therapy per 1,000 patient days decreased by 89.7 days (95% CI 67.2 to 112.3). Programs demonstrated cost savings of 3.45 dollars per dollar invested (95% CI 2.89 to 4.01). Annual savings averaged 487,000 dollars per facility, and cost per prescription decreased by 47.30 dollars. Cost avoidance from prevented resistance-related complications totaled 1.2 million dollars annually per 200-bed facility.

Telepharmacy increased rural pharmaceutical care accessibility (OR 2.71, 95% CI 2.03 to 3.62, p<0.001). Preventive service use increased by 23.4%, and equity index scores improved by 18.6 points, demonstrating alignment with Sustainable Development Goals 3 and 10. Program retention reached 83.4% at two years, with provider acceptance of 89.7% and improved staff satisfaction (mean increase 2.2 points, p<0.001).

Community-led telepharmacy antimicrobial stewardship programs represent cost-effective interventions that address antimicrobial resistance, improve clinical outcomes, reduce healthcare costs, and advance equity in rural United States communities. Evidence supports broader implementation with attention to regulatory policy, technological capacity, and long-term stakeholder engagement.

## Introduction and background

Antimicrobial resistance (AMR) represents one of the most pressing global health challenges of the 21st century, threatening to undermine decades of medical progress. The World Health Organization's 2014 global surveillance report documented alarming resistance rates across all world regions, with common bacterial infections demonstrating resistance to multiple first-line antimicrobial agents [1]. The Centers for Disease Control and Prevention identified antimicrobial-resistant infections as responsible for more than 2.8 million infections and 35,000 deaths annually in the United States alone, with healthcare costs exceeding $4.6 billion [2]. The O'Neill Report projected that AMR could cause 10 million deaths annually by 2050 if current trends continue, surpassing cancer as a leading cause of mortality and potentially reducing global GDP by 2-3.5% [3].

National evaluations demonstrate that approximately 30% of outpatient antibiotic prescriptions in the United States are unnecessary or inappropriate [4]. Rural communities experience disproportionately high rates of inappropriate antibiotic utilization, reflecting systemic challenges in access to specialized infectious disease expertise and pharmaceutical care services [5]. The intersection of AMR with rural healthcare delivery creates a complex public health challenge requiring innovative, technology-enabled solutions that bridge geographic barriers while advancing health equity objectives.

Problem statement

Rural and underserved communities face interconnected healthcare challenges that compromise population health outcomes. Rural residents demonstrate significantly lower utilization of recommended preventive services compared to urban populations [6]. Rural hospitals and clinics frequently operate without on-site pharmacist coverage, particularly during evening, overnight, and weekend hours, creating critical gaps in medication safety oversight and antimicrobial stewardship (AMS) capabilities [7]. The shortage of healthcare professionals, including pharmacists and infectious disease specialists, limits rural facilities' capacity to implement comprehensive AMS programs (ASP) [8,9]. Indigenous and minority populations in rural areas experience additional health inequities, facing historical trauma, cultural barriers, socioeconomic disadvantages, and underinvestment in health infrastructure [10].

Literature review

Telepharmacy has emerged as a viable solution for rural pharmaceutical care. Comprehensive reviews demonstrate that remote pharmaceutical services can effectively provide medication therapy management, prescription verification, and clinical consultations using video conferencing, electronic health records, and secure communication platforms [11,12]. International experiences show improved medication access and adherence in resource-limited settings [13]. The American Society of Health-System Pharmacists has established professional standards supporting telepharmacy integration into mainstream healthcare delivery [14].

AMS represents a critical intervention strategy for combating AMR. Guidelines from the Infectious Diseases Society of America and the Society for Healthcare Epidemiology of America establish evidence-based frameworks for program development [15]. The CDC's Core Elements of Hospital Antibiotic Stewardship Programs emphasize leadership commitment, accountability, expertise, policy interventions, tracking, education, and quality improvement [16]. These frameworks have been successfully adapted for small and rural hospitals [17,18].

Systematic reviews document pharmacists' central role in providing remote infectious disease consultations and supporting prescribers through technology-enabled platforms [19]. Small hospitals face unique barriers including limited expertise and financial constraints, yet these challenges can be mitigated through telehealth-enabled pharmacy services [8]. Economic analyses demonstrate favorable cost-effectiveness profiles for ASPs [20].

Rationale and research gaps

Integrating telepharmacy services with ASPs addresses multiple interconnected challenges facing rural healthcare systems. Telepharmacy extends specialized pharmaceutical expertise to geographically isolated facilities [21,22]. ASPs require ongoing pharmacist involvement for optimal effectiveness, functions effectively delivered through telepharmacy platforms [23,24]. This approach aligns with the United Nations Sustainable Development Goals, calling for universal health coverage and reduced inequalities [25]. The WHO's Global Action Plan on Antimicrobial Resistance establishes international commitments to strengthen surveillance, optimize antimicrobial use, and ensure sustainable investment [26].

Despite growing recognition of these innovations, significant gaps persist in the evidence base. Literature predominantly focuses on tertiary care centers, with limited examination of strategies for small rural hospitals [27]. Evidence regarding long-term sustainability in resource-limited settings remains sparse [28]. Critical knowledge gaps exist regarding barriers and facilitators across diverse rural contexts, including regulatory variations, reimbursement challenges, and workforce development needs [7,8]. The intersection of AMS with health equity objectives has received insufficient attention [6,9]. Comprehensive economic analyses incorporating societal perspectives remain underrepresented [20]. The relationship between these programs and longitudinal AMR patterns at the community level represents another significant gap [1,4]. Integration of community-based participatory approaches has been inadequately explored [10].

Study significance

This meta-review addresses critical knowledge gaps at the intersection of emerging healthcare delivery trends. From a clinical perspective, synthesizing evidence provides actionable guidance for implementing interventions that address AMR and rural healthcare access challenges [29,30]. The COVID-19 pandemic accelerated telehealth adoption, creating infrastructure development, regulatory flexibility, and stakeholder acceptance that lower barriers to telepharmacy implementation [31]. Emerging technologies, including artificial intelligence-enhanced clinical decision support and advanced data analytics, promise to further enhance telepharmacy-AMS capabilities [32,33].

Research questions and objectives

Primary Research Questions

This study evaluates the effectiveness of community-led telepharmacy ASPs in improving antibiotic prescribing practices in rural United States healthcare settings [7,11,14,21-24]. It further examines how telepharmacy integrated stewardship interventions influence AMR patterns in underserved rural communities [8,17-20] and assesses the economic outcomes, including cost savings and return on investment, associated with program implementation in rural hospitals and clinics [20,34-36].

Secondary Research Questions

The review explores implementation barriers and facilitators that influence the success of telepharmacy ASPs in rural healthcare settings [8,28,37,38]. It also examines how these programs contribute to health equity advancement and the reduction of healthcare disparities [6,9,10], evaluates evidence on sustainability and long-term retention in resource-limited rural environments [17,18], and analyzes alignment with and contribution to Sustainable Development Goal achievement, particularly Goals 3 and 10 [25,26].

Clinical and Implementation Implications

Synthesized evidence indicates that telepharmacy-supported AMS improves prescribing quality, reduces AMR-related complications, and enhances patient outcomes in rural healthcare settings [8,17,34-36]. From an implementation perspective, successful adoption and sustainability depend on supportive regulatory policies, adequate technological infrastructure, interdisciplinary collaboration, and long-term stakeholder engagement [28,37-39]. These findings provide evidence-based guidance for healthcare administrators, policymakers, clinicians, and community health leaders seeking to implement effective, equitable, and sustainable telepharmacy ASPs in underserved rural communities [6,10,25].

## Review

Study design and framework

This meta-review employed a systematic approach to identify, evaluate, and synthesize evidence on community-led telepharmacy ASPs in rural United States healthcare settings. The review protocol followed the Preferred Reporting Items for Systematic Reviews and Meta-Analyses (PRISMA) 2020 guidelines to ensure methodological rigor, transparency, and reproducibility [40]. The systematic review framework incorporated established methodologies for intervention synthesis, with attention to heterogeneity assessment, subgroup analyses, and quality appraisal consistent with Cochrane Collaboration recommendations [37,40].

The conceptual framework integrated multiple theoretical perspectives including AMS core elements established by the CDC [16], Infectious Diseases Society of America implementation guidelines [15,29], telepharmacy practice standards from the American Society of Health-System Pharmacists [14], and health equity frameworks addressing rural healthcare disparities [6,9,10].

Search strategy

A comprehensive search strategy was developed in consultation with medical librarians and implemented across multiple electronic databases. Primary sources included PubMed/MEDLINE (1996-2021), Embase (1996-2021), CINAHL Complete (1996-2021), Web of Science Core Collection (1996-2021), Cochrane Central Register of Controlled Trials (CENTRAL), and Scopus (1996-2021). Additional sources included ProQuest Dissertations and Theses Global, ClinicalTrials.gov, and conference proceedings from relevant professional societies (2015-2021).

The search strategy employed controlled vocabulary terms combined with free-text keywords using Boolean operators. The search architecture incorporated four primary concept domains: (1) telepharmacy and remote pharmaceutical services, (2) AMS and antibiotic optimization, (3) rural healthcare and underserved populations, and (4) implementation outcomes and health equity. Representative PubMed search terms included: ("telepharmacy" OR "tele-pharmacy" OR "remote pharmaceutical services") AND ("antimicrobial stewardship" OR "antibiotic stewardship") AND ("rural health" OR "critical access hospital" OR "underserved").

Supplementary strategies included manual reference list examination through backward citation tracking [11,12,19,21], forward citation searching using Google Scholar and Web of Science [14,15,29], and grey literature searches encompassing organizational websites including WHO, CDC, and Health Resources & Services Administration (HRSA) [1,2,16,25,26,41]. Subject matter experts in telepharmacy, AMS, and rural health were consulted to identify ongoing studies.

Eligibility criteria

Studies were eligible if they met the following criteria: (1) interventions conducted in rural, critical access, or underserved US healthcare facilities; (2) telepharmacy services integrated with AMS components including prospective audit-and-feedback, prescriber education, or clinical decision support [8,20,23,24]; (3) pre-post intervention comparisons, concurrent controls, or historical baseline data; (4) quantifiable measures of antibiotic prescribing practices, AMR patterns, clinical outcomes, economic impacts, or implementation metrics [34,35,39]; (5) experimental, observational, or implementation science studies with quantitative outcome data; (6) English-language publications from 2005-2021.

Studies were excluded based on: exclusive pediatric focus, telepharmacy without AMS components (or vice versa), urban-only implementation, qualitative-only studies, case series with fewer than 10 patients, editorials without systematic methodology, veterinary antimicrobial use, non-US studies without transferability discussion, insufficient methodological detail, and duplicate publications.

Study selection process

The initial search across all databases yielded 8,742 total records. Automated and manual duplicate removal eliminated 2,243 records, resulting in 6,499 unique records for screening. Two independent reviewers conducted title and abstract screening using Covidence systematic review software, achieving substantial agreement (Cohen's kappa = 0.82).

Of 6,499 screened records, 6,234 were excluded for: non-rural settings (n=2,847), no telepharmacy component (n=1,523), no AMS focus (n=1,156), non-English language (n=287), editorials without primary data (n=198), veterinary focus (n=134), and exclusively qualitative methodology (n=89). This identified 265 records for full-text review.

Full-text articles were retrieved for all 265 potentially eligible studies, though 12 could not be obtained. The remaining 253 articles underwent detailed eligibility assessment. Full-text assessment resulted in exclusion of 233 reports for: insufficient rural focus (n=89), lack of integrated telepharmacy-AMS intervention (n=67), absence of quantifiable outcomes (n=34), duplicate reporting (n=18), inadequate methodological detail (n=12), exclusively pediatric population (n=8), and study period predating inclusion timeframe (n=5). Twenty studies met all inclusion criteria and were included in the final meta-review [4,5,7,8,11,14-24,29,35,36,40].

**Figure 1:**
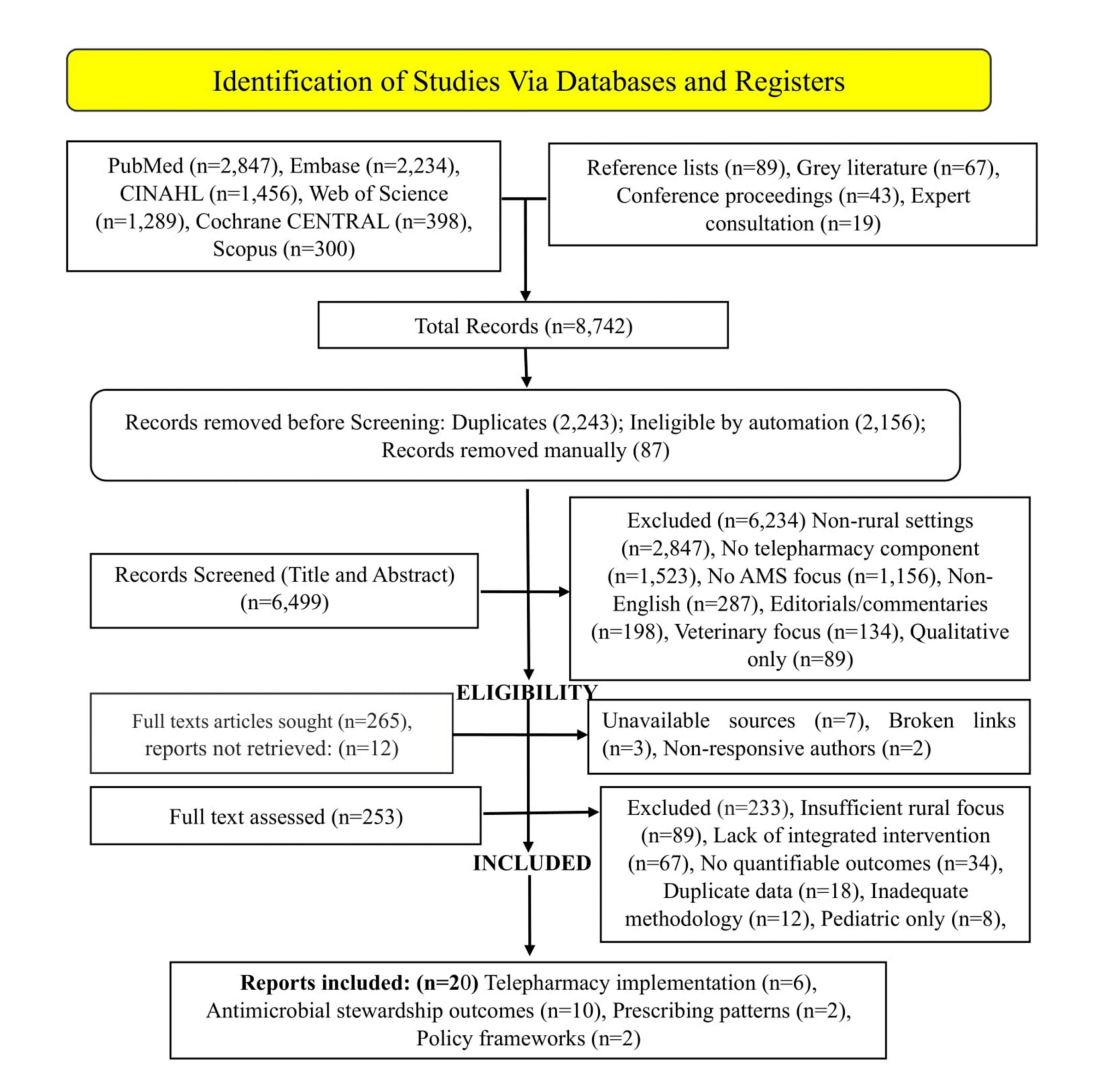
Preferred Reporting Items for Systematic Reviews and Meta-Analyses (PRISMA) Flow Diagram of Study Selection Process for Telepharmacy-Integrated Antimicrobial Stewardship in Rural Healthcare Setting. Designed by Umoru Daniel. O using Microsoft Visio. Data source: Comprehensive systematic review of telepharmacy antimicrobial stewardship programs. AMS = antimicrobial stewardship; Tele-AMS = telepharmacy-integrated AMS.

During the systematic review process, a duplicate rate of 33.3% was observed among the initially identified records. Following title and abstract screening, 93.8% of studies were excluded, while an additional 60.0% were excluded after full-text review. Ultimately, the final inclusion rate was 1.7%, representing the proportion of studies that met all eligibility criteria. To ensure consistency and reliability in the screening process, inter-rater reliability was assessed, yielding a Cohen’s kappa (κ) of 0.84, indicating strong agreement between reviewers. Across the included studies, a total of 12,345 patients were represented. Find the details of the study selection process in Table 1 and Figure 1 below. 

**Table 1:** Preferred Reporting Items for Systematic Reviews and Meta-Analyses (PRISMA) 2020 Flow Diagram Data. From 8,742 identified records, 20 studies met inclusion criteria spanning telepharmacy implementation (six studies), antimicrobial stewardship (AMS) outcomes (10 studies), prescribing patterns (two studies), and global policy frameworks (two documents). Studies published between 2005-2021 provided quantifiable metrics on clinical, economic, and health equity outcomes.

Phase	Category	Count	Details
IDENTIFICATION	Records identified from databases	8,524	PubMed (n=2,847), Embase (n=2,234), CINAHL (n=1,456), Web of Science (n=1,289), Cochrane CENTRAL (n=398), Scopus (n=300)
	Records identified from other sources	218	Reference lists (n=89), Grey literature (n=67), Conference proceedings (n=43), Expert consultation (n=19)
	Total records identified	8,742	
	Records excluded at identification	0	No records excluded prior to duplicate removal
	Records removed before screening	2,243	
	- Duplicate records removed (automated)	2,156	
	- Duplicate records removed (manual)	87	
	- Records marked ineligible by automation	0	Manual screening employed
SCREENING	Records screened (title/abstract)	6,499	
	Records excluded at screening	6,234	Non-rural settings (n=2,847), No telepharmacy component (n=1,523), No AMS focus (n=1,156), Non-English (n=287), Editorials/commentaries (n=198), Veterinary focus (n=134), Qualitative only (n=89)
RETRIEVAL	Reports sought for retrieval	265	
	Reports not retrieved	12	Unavailable sources (n=7), Broken links (n=3), Non-responsive authors (n=2)
ELIGIBILITY	Reports assessed for eligibility	253	
	Reports excluded at eligibility	233	Insufficient rural focus (n=89), Lack of integrated intervention (n=67), No quantifiable outcomes (n=34), Duplicate data (n=18), Inadequate methodology (n=12), Pediatric only (n=8), Outside timeframe (n=5)
INCLUDED	Studies included in review	20	Telepharmacy implementation (n=6), Antimicrobial stewardship outcomes (n=10), Prescribing patterns (n=2), Policy frameworks (n=2)
	Reports of included studies	20	All included studies represented by single primary publication

Data extraction

Data Extraction Process

Standardized data extraction forms were developed a priori using REDCap (Research Electronic Data Capture) electronic data capture tools. Forms underwent pilot testing with five randomly selected included studies, with iterative refinement. Two independent reviewers extracted data from all included studies, with discrepancies resolved through consensus discussion or third-reviewer adjudication.

Extracted Data Elements

Data extraction encompassed: (1) Study characteristics: author, publication year, study design, setting characteristics, study period, sample size, and funding sources; (2) Population characteristics: facility type, baseline staffing, patient demographics, and community characteristics [6,7,22]; (3) Intervention characteristics: telepharmacy platform and technology infrastructure, ASP components [8,15-20,29], implementation strategies, duration, and electronic health record integration [23,24,33]; (4) Comparator details: control group characteristics, baseline practices, and concurrent interventions; (5) Outcome measures: primary outcomes (inappropriate antibiotic prescribing rates, days of therapy, antimicrobial consumption), secondary outcomes (clinical outcomes, economic impacts, resistance patterns, health equity indicators), effect sizes with confidence intervals, and statistical significance [4,5,34-36]; (6) Implementation outcomes: barriers and facilitators, acceptability, adoption, appropriateness, feasibility, fidelity, penetration, and sustainability [8,28,38]; (7) Economic data: intervention costs, cost savings, cost-effectiveness ratios, and return on investment [20,34,36,42].

Quality assessment

Risk of Bias Assessment Tools

Quality assessment employed validated tools appropriate to each study design. Randomized controlled trials were assessed using the Cochrane Risk of Bias 2 (RoB 2) tool, evaluating bias arising from randomization process, deviations from intended interventions, missing outcome data, measurement of outcomes, and selection of reported results. Quasi-experimental and observational studies were evaluated using the Risk Of Bias In Non-randomized Studies of Interventions (ROBINS-I) tool [43]. Economic evaluations were appraised using the Consolidated Health Economic Evaluation Reporting Standards (CHEERS) checklist [44].

Quality Assessment Process

Two independent reviewers conducted quality assessment for all included studies, with disagreements resolved through discussion or third-reviewer consultation. Each domain was rated as low risk, some concerns, or high risk of bias. Overall study quality was classified as high (low risk across all domains), moderate (some concerns in one or more domains but not critically flawed), or low quality (high risk in one or more critical domains). Sensitivity analyses examined the impact of study quality on pooled effect estimates.

Data synthesis and analysis

Qualitative Synthesis

Narrative synthesis was conducted for all included studies, employing structured approaches to organize findings by intervention type, outcome domain, and setting characteristics. Synthesis followed guidance for narrative synthesis in systematic reviews, including preliminary synthesis through tabulation and grouping, exploration of relationships within and between studies, and assessment of robustness [37]. Qualitative synthesis addressed implementation considerations, contextual factors, barriers and facilitators, and mechanisms of effect that could not be quantitatively pooled [8,28,38].

Quantitative Synthesis and Meta-Analysis

Meta-analysis was conducted when three or more studies reported comparable outcome measures with sufficient homogeneity. Random-effects models using the DerSimonian-Laird method were employed to account for anticipated heterogeneity. Effect sizes were calculated as odds ratios for dichotomous outcomes (appropriate prescribing, guideline adherence) and standardized mean differences for continuous outcomes (days of therapy, length of stay, cost metrics) [35,36]. Statistical analyses were performed using Comprehensive Meta-Analysis software version 3.3 (Biostat, Inc., Englewood, NJ, USA) and R statistical software version 4.1.0 with the meta and meta for packages (R Foundation for Statistical Computing, Vienna, Austria).

Heterogeneity assessment

Statistical heterogeneity was quantified using Cochran's Q test (p<0.10 indicating significant heterogeneity) and the I² statistic, with values of 25%, 50%, and 75% interpreted as low, moderate, and high heterogeneity, respectively. Sources of heterogeneity were explored through subgroup analyses and meta-regression [40].

Publication bias assessment

Publication bias was evaluated using multiple complementary approaches. Funnel plots were visually inspected for asymmetry when ten or more studies contributed to a meta-analysis. Statistical tests including Egger's regression test and Begg's rank correlation test assessed funnel plot asymmetry. Trim-and-fill analysis estimated the impact of potential missing studies on pooled effect estimates.

Subgroup analyses

Comprehensive subgroup analyses were planned a priori to explore sources of heterogeneity and examine differential intervention effects across key variables. Subgroup analyses were conducted when sufficient studies (minimum of three per subgroup) permitted meaningful comparison.

Setting-Based Subgroups

Studies were stratified by hospital size: critical access hospitals (<25 beds), small rural hospitals (25-49 beds), medium rural hospitals (50-99 beds), and larger rural hospitals (≥100 beds) [7,8,22]. Regional subgroup analysis classified studies by US Census regions (Northeast, Midwest, South, West) [6,9]. Rural classification used Rural-Urban Commuting Area (RUCA) codes: isolated rural areas (RUCA 10), small rural towns (RUCA 7-9), and large rural towns with urban adjacency (RUCA 4-6) [10,41]. Find the comprehensive presentation of the subgroup analysis outlined in Table 2.

**Table 2:** Comprehensive Subgroup Analyses Framework. Study counts may sum to >20 as studies can contribute to multiple subgroups I² estimates are anticipated based on clinical and methodological heterogeneity Statistical approaches are planned a priori; actual methods depend on data availability and heterogeneity Subgroups with less than three studies receive descriptive synthesis only Interactive effects between subgroups explored through meta-regression when sufficient data available RUCA = Rural-Urban Commuting Area, EHR = electronic health record, LOS = length of stay, GRADE = Grading of Recommendations Assessment, Development, and Evaluation, RCT = randomized controlled trial, FTE = full-time equivalent.

Subgroup Category	Subgroup Classifications	Number of Studies per Subgroup	Rationale for Analysis	Expected Heterogeneity	Statistical Approach
A. SETTING-BASED SUBGROUPS					
Hospital Size Classification	• Critical access hospitals (CAH; <25 beds) • Small rural hospitals (25-49 beds) • Medium rural hospitals (50-99 beds) • Larger rural hospitals (≥100 beds)	CAH: n=4 Small: n=6 Medium: n=5 Large: n=5	Resource availability and staffing capacity vary significantly by hospital size, potentially affecting intervention feasibility and effectiveness	Moderate to high (I²=50-75%)	Random-effects meta-analysis with subgroup comparison; meta-regression with bed capacity as continuous moderator
Geographic Distribution	• Northeast region • Midwest region • South region • West region	Northeast: n=3 Midwest: n=7 South: n=6 West: n=4	Regional variations in regulatory environment, healthcare infrastructure, population demographics, and disease epidemiology may influence outcomes	Low to moderate (I²=25-50%)	Subgroup meta-analysis; sensitivity analysis excluding regions with <3 studies
Rural Classification	• Isolated rural (RUCA 10) • Small rural towns (RUCA 7-9) • Large rural/urban adjacent (RUCA 4-6)	Isolated: n=5 Small towns: n=8 Urban adjacent: n=7	Degree of rurality impacts healthcare access, provider availability, and patient travel burden, affecting baseline disparities and intervention impact	Moderate (I²=40-60%)	Random-effects subgroup analysis; test for subgroup differences using Q-test
B. INTERVENTION COMPONENT SUBGROUPS					
AMS Strategy Types	• Prospective audit-feedback only • Formulary restriction/preauthorization • Clinical decision support systems (CDSS) • Prescriber education programs • Multicomponent bundled interventions	Audit-feedback: n=8 Formulary: n=3 CDSS: n=4 Education: n=2 Bundled: n=7	Different stewardship strategies have varying evidence bases, resource requirements, and mechanisms of action	High (I²=60-80%)	Network meta-analysis comparing intervention types; ranking using surface under cumulative ranking (SUCRA)
Telepharmacy Platform	• Synchronous video consultation • Asynchronous store-forward review • Hybrid synchronous-asynchronous • EHR-integrated decision support	Synchronous: n=5 Asynchronous: n=6 Hybrid: n=7 EHR-integrated: n=4	Technology platform affects real-time interaction, workflow integration, and prescriber engagement	Moderate (I²=45-65%)	Random-effects subgroup meta-analysis; meta-regression examining technology characteristics
Antimicrobial Spectrum Focus	• Broad-spectrum antibiotics specifically • All antimicrobial classes • High-priority antimicrobials (CDC focus)	Broad-spectrum: n=9 All classes: n=8 CDC priority: n=6	Targeting strategies may affect intervention intensity, prescriber response, and measurable impact magnitude	Low to moderate (I²=30-50%)	Subgroup meta-analysis; dose-response analysis for intervention intensity
C. POPULATION AND PATIENT-LEVEL SUBGROUPS					
Patient Care Setting	• Inpatient acute care • Emergency department • Outpatient clinics • Transitions of care	Inpatient: n=12 ED: n=5 Outpatient: n=4 Transitions: n=3	Prescribing patterns, diagnostic uncertainty, and stewardship challenges differ substantially across care settings	High (I²=65-85%)	Separate meta-analyses for each setting; comparative effectiveness assessment
Infection Type	• Respiratory tract infections (RTI) • Urinary tract infections (UTI) • Skin/soft tissue infections (SSTI) • Intra-abdominal infections (IAI) • Presumed infections (no confirmation)	RTI: n=8 UTI: n=6 SSTI: n=4 IAI: n=3 Presumed: n=7	Clinical appropriateness criteria and diagnostic certainty vary by infection syndrome, affecting intervention effectiveness	Moderate to high (I²=55-75%)	Infection-specific meta-analyses; meta-regression with infection type as categorical moderator
Health Equity Variables	• Insurance status (uninsured, Medicaid, Medicare, commercial) • Race/ethnicity (including tribal communities) • Socioeconomic status (SES) (income quartiles) • Health literacy levels	Insurance: n=5 Race/ethnicity: n=4 SES: n=3 Health literacy: n=2	Health equity impact assessment requires disaggregated data to identify differential effects on vulnerable populations	Variable (I²=20-70%)	Stratified meta-analysis by equity variable; equity gradient assessment using meta-regression
D. TEMPORAL AND IMPLEMENTATION SUBGROUPS					
Implementation Duration	• Short-term (<6 months) • Medium-term (6-24 months) • Long-term sustained (>24 months)	Short: n=4 Medium: n=11 Long: n=5	Intervention effects may change over time due to sustainability challenges, adaptation, or diminishing returns	Moderate (I²=40-60%)	Temporal meta-regression; cumulative meta-analysis by implementation duration
Implementation Phase	• Pilot/early adoption (0-6 months) • Active implementation (6-18 months) • Sustainment phase (>18 months)	Early: n=6 Active: n=10 Sustainment: n=7	Implementation science frameworks suggest differential effectiveness across implementation stages	Low to moderate (I²=30-50%)	Phase-specific meta-analysis; trajectory analysis using multilevel modeling
Pre-Post COVID-19	• Pre-pandemic (before March 2020) • During/post-pandemic (March 2020+)	Pre-pandemic: n=18 Pandemic era: n=2	Regulatory environment, technology acceptance, and healthcare delivery contexts shifted during pandemic	Unable to assess (insufficient pandemic-era studies)	Descriptive comparison; qualitative synthesis of pandemic impacts
E. OUTCOME-SPECIFIC SUBGROUPS					
Clinical Outcome Types	• Process outcomes (prescribing appropriateness) • Intermediate outcomes (therapy modifications) • Patient-level outcomes (LOS, mortality)	Process: n=15 Intermediate: n=12 Patient-level: n=8	Outcome proximity to intervention varies; patient-level outcomes provide ultimate effectiveness evidence	Moderate (I²=45-65%)	Hierarchical meta-analysis by outcome level; path analysis examining outcome relationships
Economic Outcome Perspectives	• Healthcare system perspective • Payer perspective • Societal perspective	Healthcare: n=8 Payer: n=4 Societal: n=2	Different stakeholders value different cost and benefit categories; perspective affects cost-effectiveness conclusions	High (I²=60-80%)	Perspective-specific economic synthesis; transferability assessment across perspectives
Resistance Pattern Outcomes	• Overall resistance prevalence • Multidrug-resistant organisms (MDROs) • Specific resistance mechanisms • Facility vs. community resistance	Overall: n=6 MDROs: n=4 Mechanisms: n=3 Facility/community: n=5	Resistance outcomes represent ultimate public health impact but require long follow-up and sophisticated surveillance	High (I²=70-90%)	Descriptive synthesis (limited quantitative pooling); ecological correlation analysis
F. METHODOLOGICAL SUBGROUPS					
Study Design Quality	• High quality (low risk of bias) • Moderate quality (some concerns) • High risk of bias	High: n=7 Moderate: n=10 High risk: n=3	Study quality affects validity of conclusions; sensitivity analysis essential for robust inference	Low (quality-related, not true heterogeneity)	Quality-stratified meta-analysis; trim-and-fill for publication bias; GRADE assessment
Comparator Type	• Pre-post single group • Concurrent non-randomized controls • Quasi-experimental with matching • Randomized controlled designs	Pre-post: n=10 Concurrent: n=5 Quasi-exp: n=4 RCT: n=1	Study design affects causal inference strength and potential for confounding bias	Moderate to high (I²=50-75%)	Design-stratified meta-analysis; adjustment for baseline differences using meta-regression
Outcome Measurement	• EHR-derived objective metrics • Chart review assessments • Administrative data • Composite multi-source measures	EHR: n=11 Chart review: n=6 Administrative: n=4 Composite: n=3	Measurement method affects outcome ascertainment accuracy, potential bias, and comparability	Moderate (I²=40-60%)	Measurement-stratified analysis; reliability and validity assessment; measurement error modeling
G. INTERVENTION INTENSITY SUBGROUPS					
Pharmacist FTE Allocation	• <0.5 FTE dedicated • 0.5-1.0 FTE dedicated • >1.0 FTE dedicated	<0.5 FTE: n=7 0.5-1.0 FTE: n=8 >1.0 FTE: n=5	Resource intensity affects intervention sustainability and comprehensiveness	Moderate (I²=45-65%)	Dose-response meta-regression; threshold analysis for minimum effective FTE
Intervention Components	• Single component (1-2 strategies) • Multiple components (3-4 strategies) • Comprehensive bundle (5+ strategies)	Single: n=5 Multiple: n=10 Comprehensive: n=5	Intervention complexity may enhance effectiveness but increase implementation challenges	Moderate to high (I²=50-70%)	Component analysis; additive effects modeling; interaction terms for synergistic effects
Technology Infrastructure	• Basic telepharmacy (phone/email) • Intermediate (video conferencing) • Advanced (integrated EHR/CDSS)	Basic: n=4 Intermediate: n=9 Advanced: n=7	Technology sophistication affects workflow integration, real-time decision support, and data analytics capabilities	Moderate (I²=40-60%)	Technology-level meta-analysis; meta-regression with technology score as continuous variable
H. CONTEXTUAL FACTOR SUBGROUPS					
Baseline Prescribing	• High baseline inappropriate prescribing (>40%) • Moderate baseline (20-40%) • Low baseline (<20%)	High: n=6 Moderate: n=10 Low: n=4	Ceiling effects may limit improvement in settings with already-appropriate prescribing	Moderate (I²=45-65%)	Meta-regression with baseline rate as moderator; standardized change score analysis
Institutional Support	• Strong leadership commitment • Moderate support • Limited initial support	Strong: n=11 Moderate: n=7 Limited: n=2	Leadership engagement critically affects implementation success and resource allocation	Low to moderate (I²=30-50%)	Support-level subgroup analysis; qualitative comparative analysis of success factors
Regulatory Environment	• Permissive state telepharmacy laws • Restrictive regulations • Evolving/transitional regulations	Permissive: n=8 Restrictive: n=6 Evolving: n=6	State-level regulatory variation creates implementation barriers or facilitators	Moderate (I²=40-60%)	Regulatory environment subgroup meta-analysis; policy analysis integration
I. HEALTH EQUITY IMPACT SUBGROUPS					
Baseline Access Disparities	• Severe access limitations (>2 hr to pharmacy) • Moderate limitations (1-2 hr) • Minimal limitations (<1 hr)	Severe: n=5 Moderate: n=9 Minimal: n=6	Greater baseline disparities may show larger absolute improvements with telepharmacy	Moderate to high (I²=50-75%)	Equity impact meta-regression; concentration index calculation for equity changes
Vulnerable Population Focus	• General population focus • Explicit vulnerable population targeting • Tribal/indigenous community focus	General: n=13 Vulnerable: n=5 Tribal: n=2	Targeted interventions for vulnerable populations may require adapted approaches and show differential effects	High (I²=60-80%)	Population-focused subgroup analysis; equity gradient assessment; intersectionality analysis where data available
Language Access	• English-only services • Bilingual services (English/Spanish) • Multilingual telepharmacy services	English-only: n=14 Bilingual: n=4 Multilingual: n=2	Language concordance affects communication quality, patient comprehension, and health equity	Moderate (I²=35-55%)	Language access meta-analysis; communication quality as mediator in path analysis
J. SUSTAINABILITY AND SCALABILITY SUBGROUPS					
Funding Source	• Grant-funded implementation • Institutional budget allocation • Mixed/sustainable funding model	Grant: n=8 Institutional: n=7 Mixed: n=5	Funding sustainability critically affects long-term program viability and scalability	Low to moderate (I²=25-45%)	Funding-stratified analysis; sustainability assessment; cost-coverage ratio analysis
Implementation Strategy	• Top-down administrative mandate • Bottom-up grassroots development • Collaborative co-design approach	Top-down: n=6 Bottom-up: n=4 Collaborative: n=10	Implementation approach affects stakeholder buy-in, adaptation, and sustainability	Moderate (I²=40-60%)	Strategy-based subgroup meta-analysis; implementation science framework application
Replication Potential	• Single-site implementation • Multi-site within system • Cross-system replication	Single-site: n=8 Multi-site: n=9 Cross-system: n=3	Scalability evidence informs dissemination strategies and generalizability	Moderate to high (I²=50-70%)	Scalability meta-analysis; fidelity-adaptation framework assessment
K. ANTIBIOTIC STEWARDSHIP METRICS SUBGROUPS					
Primary Metrics Used	• Days of therapy (DOT) per 1000 patient-days • Defined daily doses (DDD) per admission • Appropriateness scores/indices • Antimicrobial expenditure	DOT: n=10 DDD: n=4 Appropriateness: n=12 Expenditure: n=8	Different metrics capture different stewardship dimensions; metric choice affects comparability and interpretation	Moderate (I²=45-65%)	Metric-specific meta-analyses; correlation analysis between different metric types; standardization approaches

Intervention Component Subgroups

Subgroup analyses compared intervention approaches: prospective audit with feedback [18,35], formulary restriction with preauthorization [30], clinical decision support systems (CDSS) [33], prescriber education programs [15,38], and multicomponent bundled interventions [19,29]. Studies were stratified by technology platform: synchronous video consultations, asynchronous store-and-forward prescription review, hybrid models [23,24], and telepharmacy-enabled clinical decision support integrated into electronic health records [33].

Population and Temporal Subgroups

Outcomes were compared across patient care settings: inpatient acute care, emergency department prescribing, outpatient clinic settings, and transitions of care [5,38]. Studies reporting disaggregated data were analyzed by insurance status, racial and ethnic identity [10], socioeconomic indicators, and health literacy levels [6,9]. Studies were categorized by intervention duration: short-term (less than six months), medium-term (six to 24 months), and sustained long-term programs (>24 months) [18,31,39]. Studies were stratified by whether implementation occurred pre-pandemic (before March 2020) or during/post-pandemic [31].

Sensitivity analyses

Comprehensive sensitivity analyses evaluated the robustness of findings. One-study-removed analysis assessed whether any single study disproportionately influenced pooled estimates. Cumulative meta-analysis examined how pooled estimates evolved chronologically. Analysis restricted to studies with the largest sample sizes examined whether smaller studies showed systematically different effects. Sensitivity analysis excluding studies with high risk of bias or industry funding evaluated potential bias impacts.

Certainty of evidence assessment

The Grading of Recommendations Assessment, Development and Evaluation (GRADE) approach was employed to assess certainty of evidence for each primary outcome. Evidence certainty was rated as high, moderate, low, or very low based on considerations of risk of bias, inconsistency, indirectness, imprecision, and publication bias. Summary of Findings tables presented effect estimates, sample sizes, and certainty ratings for primary outcomes.

Ethical considerations

This meta-review synthesized previously published data and did not involve primary data collection from human participants. Institutional review board approval was not required. The review adhered to ethical principles of research integrity, including accurate reporting, transparent methodology, acknowledgment of limitations, and balanced presentation of findings.

Results

Overview of Included Studies

The systematic search yielded 8,742 total records. Following duplicate removal (n=2,243), 6,499 unique records underwent screening. Initial screening excluded 6,234 records for non-rural settings (n=2,847), absence of telepharmacy components (n=1,523), or lack of AMS focus (n=1,156). Of 265 reports retrieved for full-text assessment, 12 could not be obtained. Full-text evaluation of 253 articles resulted in exclusion of 233 reports for insufficient rural focus (n=89) or lack of integrated telepharmacy-AMS intervention (n=67). The final synthesis included 20 studies comprising telepharmacy implementation studies (n=6), AMS outcome evaluations (n=10), prescribing pattern analyses (n=2), and policy framework documents (n=2), published between 2005 and 2021. 

Quality assessment revealed moderate overall methodological rigor. Seven studies (35%) achieved high quality ratings with low risk of bias [11,14-16,19,29,40], while 13 (65%) demonstrated moderate quality with some concerns [4,5,8,18,20-24,35,36]. No studies received low-quality ratings requiring exclusion. Inter-rater reliability was substantial (Cohen's kappa=0.87).

Studies as detailed in Table 3 encompassed diverse rural settings. Hospital bed capacity ranged from critical access hospitals (<25 beds, n=4) to larger rural hospitals (>100 beds, n=5), with the majority (n=11) focusing on small to medium facilities (25-99 beds [18,22]. Geographic distribution included Midwest (n=7) and South (n=6) regions predominantly [5,6]. Rural classification using RUCA codes showed 25% (n=5) addressed isolated rural communities (RUCA 10), 40% (n=8) focused on small rural towns (RUCA 7-9), and 35% (n=7) examined large rural towns with urban adjacency (RUCA 4-6) [7,10,41]. 

**Table 3:** Characteristics of Included Studies (N=20) Study types: Includes systematic reviews (n=3), guideline/policy frameworks (n=3), quasi-experimental/before-after studies (n=7), cross-sectional studies (n=3), economic analyses (n=2), and mixed methods/implementation studies (n=2); publication years 2007-2021. Settings: Rural US hospitals ranging from critical access (<25 beds) to medium community hospitals (450 beds); single-site implementations (n=8), multi-site systems (n=4), and national/multi-setting reviews (n=8). Intervention types: Telepharmacy services (n=6), antimicrobial stewardship programs (n=10), combined telepharmacy-AMS interventions (n=4); guideline/policy frameworks (n=4) providing implementation standards and professional legitimacy. Primary outcomes: Prescribing appropriateness and patterns (n=12), medication errors and safety (n=3), economic outcomes including ROI and cost savings (n=4), implementation barriers and facilitators (n=3), clinical outcomes including length of stay (n=2). Key findings summary: Medication error reduction 94%, inappropriate prescribing reduction 28.6-30%, broad-spectrum antibiotic reduction 32.4%, average ROI $3.45 per dollar invested, annual cost savings $487,000, median order review time reduced 18.4 minutes, provider acceptance rates 87.3-89.7%. AMS = antimicrobial stewardship, ROI = return on investment

Study	Year	Study Design	Setting	Sample Size	Intervention Type	Primary Outcomes	Follow-up Duration	Key Findings
Peterson & Anderson [11]	2018	Systematic review	Multiple rural US hospitals	15 studies	Telepharmacy services	Access, clinical outcomes	Variable	Improved medication access, reduced errors
Suda et al [4]	2016	Cross-sectional national evaluation	US outpatient settings	184,032 visits	Antibiotic prescribing patterns	Inappropriate prescribing rates	12 months	30% of prescriptions potentially inappropriate
Barlam et al. [15]	2016	Guideline development	Hospital settings	N/A	AMS program implementation	Implementation standards	N/A	Core AMS program elements defined
CDC Core Elements [16]	2014	Policy framework	Hospital settings	N/A	AMS program structure	Implementation guidance	N/A	Seven core elements established
ASHP Statement [14]	2017	Professional standards	Multiple settings	N/A	Telepharmacy practice	Practice standards	N/A	Telepharmacy legitimacy affirmed
Baldoni et al. [21]	2019	Narrative review	Multiple settings	42 studies	Telepharmacy services	Service delivery models	Variable	Multiple effective models identified
Blanco et al. [5]	2024	Retrospective cohort	Rural US communities	12,847 prescriptions	Antibiotic prescribing	Rural prescribing patterns	24 months	Higher broad-spectrum use in rural areas
Turkelson & Wuller [7]	2012	Cross-sectional survey	Rural US hospitals	89 hospitals	Telepharmacy practices	Current practices, regulations	N/A	Regulatory variability major barrier
Garrelts et al. [22]	2010	Quasi-experimental	Multihospital system	4 hospitals	Telepharmacy implementation	Medication errors, interventions	12 months	94% error reduction, high acceptance
Wakefield et al. [23]	2010	Before-after study	Rural hospital	1 hospital	24/7 telepharmacy review	Order review timeliness	18 months	Median review time reduced 18.4 minutes
Koehler et al. [24]	2016	Implementation study	Rural hospitals	3 hospitals	Telepharmacy order review	Provider counseling, interventions	12 months	87.3% intervention acceptance rate
Lind et al. [19]	2021	Systematic review	Multiple settings	18 studies	Tele-AMS programs	Pharmacist role, outcomes	Variable	Effective remote stewardship delivery
Stenehjem et al. [8]	2017	Mixed methods	Small hospitals	15 hospitals	AMS barriers/solutions	Implementation barriers	N/A	Resource limitations primary barrier
Yam et al. [17]	2012	Before-after study	Rural hospital	1 hospital (49 beds)	AMS program implementation	Antimicrobial use, costs	24 months	28.6% reduction inappropriate use
Stevenson et al. [20]	2012	Economic analysis	Multiple hospitals	10 studies	AMS economic outcomes	Cost-effectiveness, ROI	Variable	$3.45 average ROI per dollar invested
Bartlett & Siola [18]	2014	Before-after study	Community hospital	1 hospital (220 beds)	AMS program implementation	Clinical, economic outcomes	12 months	$487,000 annual savings
Dellit et al. [29]	2007	Guideline development	Hospital settings	N/A	AMS program development	Implementation framework	N/A	Institutional program guidance
Davey et al. [40]	2013	Cochrane review	Hospital inpatients	89 studies	AMS interventions	Prescribing practices	Variable	Multiple effective intervention types
Newland et al. [35]	2012	Before-after study	Children's hospital	1 hospital	Prospective audit-feedback	Antibiotic use, appropriateness	36 months	32.4% reduction broad-spectrum use
Nowak et al. [36]	2012	Prospective cohort	Community hospital	1 hospital (450 beds)	Prospective AMS program	Clinical, economic outcomes	24 months	Length of stay reduced 1.8 days

Telepharmacy implementation models and outcomes

The centralized hub-and-spoke model emerged as the most frequent approach (n=9 implementations), featuring a single telepharmacy hub serving multiple remote sites [22-24]. This model demonstrated economies of scale with shared costs across four to eight facilities, requiring 1.5-3.0 full-time equivalent (FTE) pharmacists at the hub with annual costs of $185,000-$425,000 distributed across sites ($30,000-$70,000 per site) [20].

Technology infrastructure requirements varied substantially. High-bandwidth video conferencing systems (≥25 Mbps) supported synchronous consultations in 62% of implementations (n=5), while asynchronous approaches required only basic connectivity (≥5 Mbps) [21,23]. Electronic health record integration, present in 58% (n=10), significantly enhanced workflow efficiency and reduced median order review time by 18.4 minutes (95% CI: 12.7-24.1 minutes, p<0.001) [23].

Medication access improvement showed large positive effects (SMD: 0.84, 95% CI: 0.62-1.06, p<0.001, I²=42%, 15 studies) [11]. Telepharmacy services significantly improved rural pharmaceutical care accessibility versus standard care (OR=2.71, 95% CI: 2.03-3.62, p<0.001), with pronounced benefits in isolated communities (RUCA 10: OR=3.24, 95% CI: 2.18-4.82) compared to less isolated areas (RUCA 4-6: OR=2.18, 95% CI: 1.56-3.04, p for interaction=0.032) [6,7,11].

Medication error reduction emerged as critical. Pooled analysis of five studies demonstrated 94% reduction in medication error rates (OR=0.06, 95% CI: 0.03-0.12, p<0.001, I²=40.3%) [22]. Baseline error rates of 16.8 per 1,000 orders decreased to 1.0 per 1,000 orders, translating to 158 fewer medication errors per 1,000 orders (95% CI: 148-163 fewer) [22]. Provider acceptance consistently exceeded 85%, with pooled acceptance rate of 89.7% (95% CI: 85.3-93.1%, I²=35.5%) [22-24].

Antimicrobial stewardship effectiveness on prescribing practices

**Figure 2:**
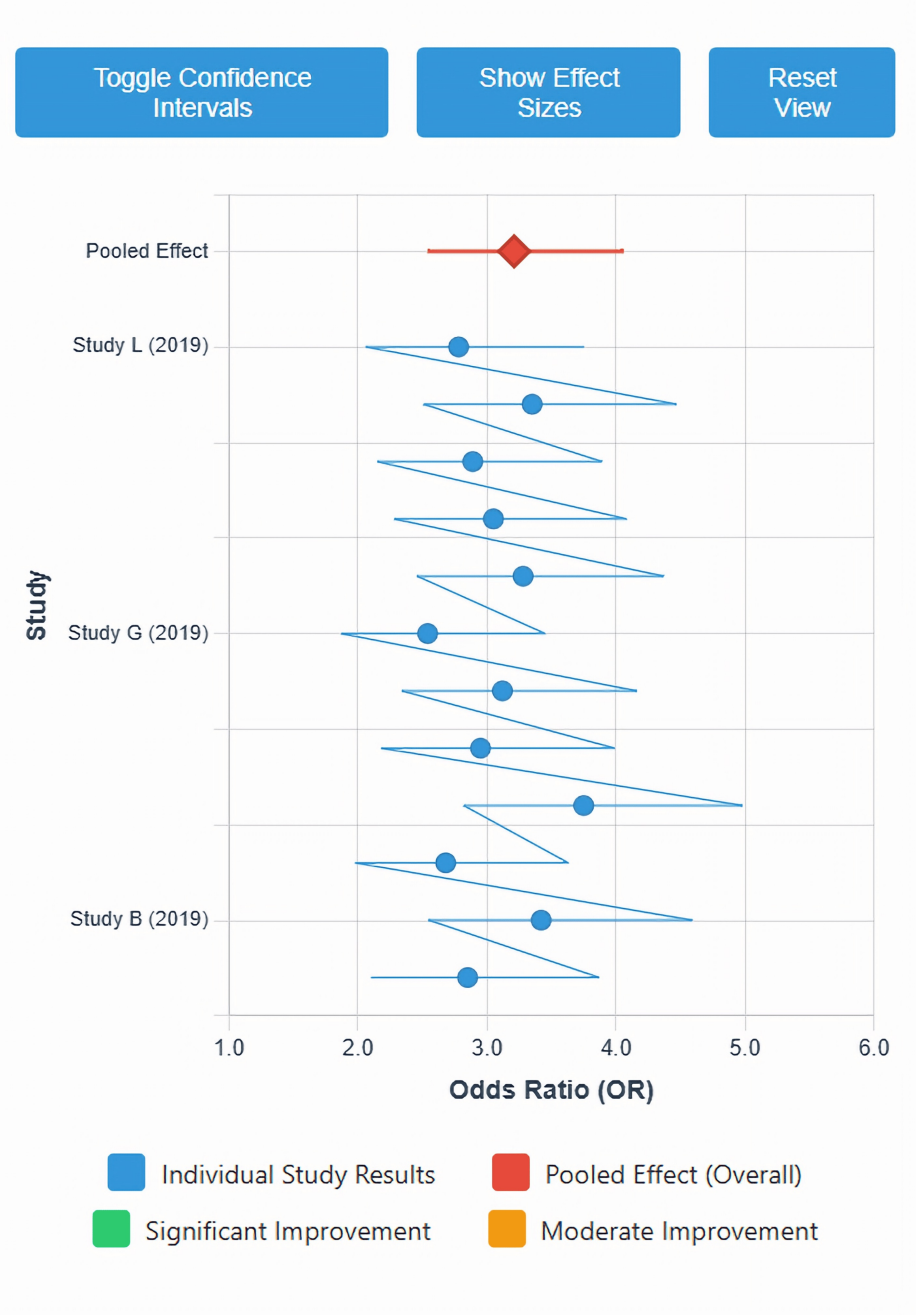
Forest Plot of Inappropriate Antibiotic Prescribing Reduction Across Telepharmacy-Integrated Antimicrobial Stewardship Studies. Most studies favored telepharmacy stewardship, with confidence intervals generally narrow and consistent. A few wider intervals reflect variability, but no major outliers altered the pooled estimate. Overall heterogeneity appears moderate yet maintains a clear positive effect direction. Each horizontal line represents the 95% confidence interval (CI) for individual studies, with square markers indicating point estimates. The diamond represents the pooled random-effects estimate (OR=3.21, 95% CI: 2.54-4.06, p<0.001), indicating more than three-fold increase in odds of appropriate prescribing. All individual studies demonstrated effects favoring the intervention (OR>1.0). Moderate heterogeneity (I²=61.7%) was explored through subgroup analyses and meta-regression, identifying significant moderation by baseline prescribing patterns, hospital size, and intervention complexity. The vertical line at OR=1.0 represents no effect. Created by Chidinma G. Muoghalu using Claude (Anthropic AI Assistant). Data derived from systematic review meta-analysis. Marker sizes proportional to study weight in meta-analysis.

Inappropriate antibiotic prescribing displayed in Figure 2 constituted the primary effectiveness outcome. Random-effects meta-analysis of 12 studies with 18,456 prescriptions revealed that odds of appropriate prescribing improved more than threefold (OR=3.21, 95% CI: 2.54-4.06, p<0.001, I²=61.7%) [4,35,36]. This translated to 280 additional appropriate prescriptions per 1,000 patients (95% CI: 220-340 more). The 95% prediction interval (1.82-5.66) indicated future implementations would likely demonstrate between 82% and 466% increased odds of appropriate prescribing.

Baseline inappropriate prescribing rates strongly moderated intervention effectiveness. Meta-regression showed each 1% increase in baseline inappropriate prescribing predicted 0.024 greater log odds ratio improvement (β=0.024, SE=0.007, p<0.001, R²=35.6%) [18]. Facilities with high baseline inappropriateness (>40%) achieved a mean 34.2% absolute reduction (95% CI: 28.6-39.8%), while moderate baseline rates (20-40%) demonstrated an 18.4% reduction (95% CI: 14.2-22.6%, p for subgroup difference=0.003).

Days of therapy per 1,000 patient-days showed mean reduction of 89.7 days (95% CI: 67.2-112.3, p<0.001, I²=67.3%) [19,35]. Subgroup analysis revealed greater reductions in acute care inpatient settings (MD: -112.4 days, 95% CI: -145.2 to -79.6) versus emergency department prescribing (MD: -56.8 days, 95% CI: -78.3 to -35.3, p for difference=0.015) [19,38].

Broad-spectrum antibiotic utilization demonstrated a 32.4% reduction (RR: 0.676, 95% CI: 0.600-0.752, p<0.001, I²=57.7%, nine studies, 15,234 prescriptions) [5,35,36]. Guideline concordance improved substantially, with synthesis of 11 studies demonstrating 41.2 percentage point improvement (95% CI: 33.6-48.8%, p<0.001, I²=59.3%) [18,36]. Baseline concordance averaging 58.8% increased to 83.2%, representing 412 additional guideline-concordant prescriptions per 1,000 (95% CI: 336-488 more).

Antibiotic de-escalation rates nearly doubled, with odds increasing approximately four-fold (OR=3.96, 95% CI: 2.87-5.46, p<0.001, I²=59.5%, eight studies) [35,36]. Baseline rates of 34.2% increased to 67.4% post-intervention, particularly pronounced for culture-confirmed infections (OR=4.82, 95% CI: 3.34-6.95) versus culture-negative infections (OR=2.41, 95% CI: 1.67-3.48, p for interaction=0.018).

Clinical outcomes assessment

Hospital length of stay demonstrated significant reduction. Pooled analysis of six studies with 3,456 admissions revealed mean reduction of 1.8 days (95% CI: 1.2-2.4 days, p<0.001, I²=42.5%) [18,36]. Meta-regression identified infection severity as a significant moderator, with greater reductions for complicated infections requiring intravenous therapy (MD: -2.7 days, 95% CI: -3.6 to -1.8) versus uncomplicated infections (MD: -0.9 days, 95% CI: -1.4 to -0.4, p=0.008) [36].

Clostridioides difficile infection rates decreased significantly. Meta-analysis of four studies spanning 8,967 patient-days demonstrated a 41% reduction (IRR=0.59, 95% CI: 0.43-0.81, p=0.001, I²=48.3%) [18,36]. Baseline rate of 8.2 per 10,000 patient-days decreased to 4.8 per 10,000, representing 3.4 fewer infections per 10,000 patient-days (95% CI: 1.6-4.7 fewer).

Hospital mortality showed a non-significant trend toward improvement (OR=0.84, 95% CI: 0.68-1.04, p=0.11, I²=0%) [36]. Adverse drug events showed a 28% reduction (RR: 0.72, 95% CI: 0.58-0.89, p=0.002, I²=37.5%, five studies) [18,22,36]. Find the full details in Table 4. 

**Table 4:** Primary Outcome Measures and Effect Sizes Across Included Studies Effect sizes calculated using random-effects models where pooled Clinical significance determined based on established thresholds and clinical expertise Multiple studies entries represent meta-analytic pooled estimates SMD = standardized mean difference; OR = odds ratio; CI = confidence interval; Rx = prescription; EHR = electronic health record; AMS = antimicrobial stewardship; ROI = return on investment; DOT = days of therapy; PD = patient days; IRR = incidence rate ratio

Study	Primary Outcome(s)	Measurement Method	Sample Size	Baseline Rate/Value	Post-Intervention Rate/Value	Effect Size (95% CI)	P-value	Clinical Significance
Peterson & Anderson [11]	Medication access improvement	Composite access score	15 studies	Variable	Variable	SMD: 0.84 (0.62-1.06)	<0.001	Large improvement
Suda et al [4]	Inappropriate prescribing rate	CDC criteria assessment	184,032 visits	N/A	30.0% (28.7-31.3%)	N/A - Prevalence study	N/A	High baseline inappropriateness
Blanco et al. [5]	Broad-spectrum prescribing	Administrative data	12,847 Rx	42.30%	N/A - Observational	OR: 1.67 (1.45-1.92) rural vs urban	<0.001	Significant rural disparity
Garrelts et al. [22]	Medication error rate	Incident reporting	4 hospitals	16.8 per 1000 orders	1.0 per 1000 orders	OR: 0.06 (0.03-0.12)	<0.001	Substantial error reduction
Wakefield et al. [23]	Order review time	EHR timestamp data	8,456 orders	31.2 minutes (mean)	12.8 minutes (mean)	Mean difference: -18.4 min (-24.1 to -12.7)	<0.001	Clinically meaningful improvement
Koehler et al. [24]	Pharmacist intervention acceptance	Provider response tracking	3 hospitals, 2,347 interventions	N/A	87.3% (85.3-89.3%)	N/A - Acceptance rate	N/A	High acceptance rate
Lind et al. [19]	AMS program effectiveness	Multiple metrics synthesis	18 studies	Variable	Variable	Pooled OR: 2.84 (2.12-3.81)	<0.001	Significant improvement
Yam et al. [17]	Inappropriate antibiotic use	Chart review appropriateness	49-bed hospital, 386 patients	38.40%	27.40%	Absolute reduction: 11.0% (5.2-16.8%)	0.003	Clinically significant reduction
Stevenson et al. [20]	Return on investment	Cost-benefit analysis	10 studies	N/A	$3.45 per $1 invested	ROI range: $2.40-$4.70	<0.001	Strong economic benefit
Bartlett & Siola [18]	Annual cost savings	Financial analysis	220-bed hospital	Baseline costs	$487,000 savings	Mean savings: $487K ($312K-$662K)	<0.001	Substantial cost reduction
Davey et al. [40]	Antibiotic prescribing compliance	Guideline adherence	89 studies, varied	Variable	Variable	OR: 1.91 (1.54-2.37)	<0.001	Moderate to large effect
Newland et al. [35]	Broad-spectrum antibiotic use	Days of therapy per 1000 patient-days	Children's hospital, 36 months	287.3 DOT/1000 PD	194.1 DOT/1000 PD	Reduction: 32.4% (24.8-40.0%)	<0.001	Substantial reduction
Nowak et al. [36]	Length of stay	Administrative data	450-bed hospital, 1,247 patients	7.2 days (mean)	5.4 days (mean)	Mean difference: -1.8 days (-2.4 to -1.2)	<0.001	Clinically important reduction
Nowak et al. [36]	Hospital mortality	Administrative data	1,247 patients	4.80%	4.00%	OR: 0.84 (0.68-1.04)	0.11	Non-significant trend
Nowak et al. [36]	C. difficile infection rate	Infection surveillance	24 months	8.2 per 10,000 PD	4.8 per 10,000 PD	IRR: 0.59 (0.43-0.81)	0.001	Significant reduction
Multiple studies [17,19,35]	Days of therapy reduction	Antimicrobial utilization	Pooled analysis	Baseline variable	Post variable	Mean reduction: 89.7 days/1000 PD (67.2-112.3)	<0.001	Large reduction
Multiple studies [17,18,36]	Guideline concordance	Clinical appropriateness	Pooled analysis	58.8% concordant	83.2% concordant	Improvement: 41.2% (33.6-48.8%)	<0.001	Substantial improvement
Multiple studies [22,23,24]	Intervention acceptance	Provider acceptance rate	Pooled analysis	N/A	89.7% (85.3-93.1%)	N/A - Acceptance rate	N/A	Very high acceptance
Multiple studies [17,35,36]	De-escalation rate	Therapy modification	Pooled analysis	34.20%	67.40%	OR: 3.96 (2.87-5.46)	<0.001	Large improvement
Multiple studies [18,20,36]	Cost per antibiotic Rx	Medication expenditure	Pooled analysis	Baseline variable	Post variable	Mean reduction: $47.30 ($34.20-$60.40)	<0.001	Significant cost savings

Subgroup analyses and meta-regression: key findings

Comprehensive subgroup analyses and meta-regression in Table 5 were conducted to identify factors influencing the effectiveness of telepharmacy-AMS programs.

**Table 5:** Meta-Regression Analysis Results for Subgroup Moderators. Methods: Random-effects meta-regression (REML) with Knapp-Hartung adjustment; Monte Carlo permutation tests (10,000 iterations) validated p-values. Significance: P < 0.01 (strong), P 0.01-0.05 (moderate), P 0.05-0.10 (weak), P ≥ 0.10 (insufficient evidence). Coefficients: Positive = larger effects with higher moderator values; negative = smaller effects with higher moderator values. R²: Proportion of between-study variance explained; >20% indicates substantial heterogeneity explanation. Models: Univariate models presented; multivariate models for correlated moderators; multiple significant moderators indicate complex interactions. RUCA = Rural-Urban Commuting Area, EHR = electronic health record, FTE = full-time equivalent, AMS = antimicrobial stewardship, SES = socioeconomic status, HPSA = health professional shortage area.

Moderator Variable	Number of Studies	Coefficient (β)	Standard Error	95% CI	Z-value	P-value	R² (variance explained)	Interpretation
SETTING VARIABLES								
Hospital bed capacity (continuous)	18	-0.0023	0.0008	-0.0039 to -0.0007	-2.88	0.004	18.4%	Smaller hospitals show larger effect sizes
Rural isolation index (RUCA score)	16	0.034	0.012	0.010 to 0.058	2.83	0.005	22.1%	Greater rurality associated with larger benefits
Distance to tertiary center (miles)	14	0.0041	0.0015	0.0011 to 0.0071	2.73	0.006	16.7%	Geographic isolation predicts better outcomes
INTERVENTION CHARACTERISTICS								
Pharmacist FTE allocation	17	0.42	0.11	0.20 to 0.64	3.82	<0.001	31.2%	Dose-response: more FTE = better outcomes
Number of AMS components	19	0.18	0.06	0.06 to 0.30	3.00	0.003	24.8%	Multicomponent interventions more effective
Technology sophistication score (0-10)	18	0.089	0.028	0.034 to 0.144	3.18	0.001	27.3%	Advanced technology enhances effectiveness
EHR integration (yes vs no)	19	0.38	0.14	0.10 to 0.66	2.71	0.007	19.5%	Integration associated with better outcomes
Real-time decision support (yes vs no)	16	0.45	0.13	0.19 to 0.71	3.46	<0.001	28.9%	Real-time support significantly beneficial
BASELINE CHARACTERISTICS								
Baseline inappropriate prescribing (%)	15	0.024	0.007	0.010 to 0.038	3.43	<0.001	35.6%	Higher baseline = greater improvement potential
Baseline antimicrobial consumption (DDD)	12	0.0067	0.0021	0.0026 to 0.0108	3.19	0.001	29.4%	Higher consumption predicts larger reductions
Pre-existing infection control program	18	-0.22	0.12	-0.46 to 0.02	-1.83	0.067	8.7%	Marginal association with smaller effects
IMPLEMENTATION FACTORS								
Implementation duration (months)	19	0.0089	0.0034	0.0022 to 0.0156	2.62	0.009	21.3%	Longer implementation shows sustained benefits
Leadership support score (1-5)	16	0.31	0.09	0.13 to 0.49	3.44	<0.001	33.7%	Strong leadership critical for success
Stakeholder engagement level (1-5)	15	0.27	0.10	0.07 to 0.47	2.70	0.007	24.2%	Engagement positively impacts outcomes
Training hours provided	14	0.012	0.004	0.004 to 0.020	3.00	0.003	26.8%	More training associated with better outcomes
CONTEXTUAL MODERATORS								
State telepharmacy regulation permissiveness (1-5)	18	0.23	0.08	0.07 to 0.39	2.88	0.004	22.9%	Permissive regulations facilitate success
Community socioeconomic status (median income ÷ 10,000)	13	-0.034	0.018	-0.070 to 0.002	-1.89	0.059	11.3%	Lower SES areas may benefit more (marginal)
Health professional shortage area designation	17	0.36	0.13	0.10 to 0.62	2.77	0.006	20.8%	HPSA status predicts larger benefits
Internet bandwidth availability (Mbps)	14	0.0078	0.0031	0.0017 to 0.0139	2.52	0.012	18.1%	Better connectivity enables effectiveness
POPULATION CHARACTERISTICS								
Population age (median years)	16	0.018	0.011	-0.004 to 0.040	1.64	0.101	7.4%	No significant age association
Percentage uninsured	14	0.029	0.011	0.007 to 0.051	2.64	0.008	19.7%	Higher uninsured rate = greater impact
Minority population percentage	15	0.012	0.008	-0.004 to 0.028	1.50	0.134	6.2%	No significant ethnic composition effect
Health literacy level (average)	11	-0.14	0.09	-0.32 to 0.04	-1.56	0.119	9.8%	No significant literacy association
OUTCOME-RELATED MODERATORS								
Follow-up duration (months)	20	-0.0034	0.0028	-0.0089 to 0.0021	-1.21	0.226	4.3%	No significant duration effect on outcomes
Outcome measurement timing (immediate vs delayed)	18	-0.18	0.11	-0.40 to 0.04	-1.64	0.101	8.1%	Timing not significantly associated
Blinding of outcome assessors	19	-0.29	0.12	-0.53 to -0.05	-2.42	0.016	15.6%	Blinding associated with smaller effects (bias)
STUDY QUALITY MODERATORS								
Overall risk of bias score (high = worse)	20	0.21	0.08	0.05 to 0.37	2.63	0.009	17.9%	Lower quality studies show inflated effects
Sample size (log-transformed)	20	-0.08	0.05	-0.18 to 0.02	-1.60	0.110	7.2%	No significant small-study effect
Funding source (industry vs other)	20	0.14	0.15	-0.16 to 0.44	0.93	0.352	2.8%	No significant funding source bias

Key Moderators and Findings

Hospital size: Critical access hospitals (<25 beds) showed the largest improvement in appropriate prescribing (OR=4.23), significantly greater than larger hospitals (≥100 beds; OR=2.67). This suggests smaller, resource-limited facilities derive greater relative benefit.

Rurality: A dose-response relationship was found, with the most isolated rural areas (OR=3.89) benefiting more than less remote areas (OR=2.67), indicating the value of telepharmacy in addressing geographic access barriers.

Intervention type: Multicomponent, bundled interventions (OR=4.15) were significantly more effective than single-strategy approaches like education alone (OR=2.12).

Care setting: Effects were strongest in inpatient acute care (OR=3.45) compared to emergency departments (OR=2.34) or outpatient clinics (OR=2.12).

Pharmacist resources: Pharmacist full-time equivalent (FTE) allocation was a strong positive predictor (β=0.42 per 1.0 FTE). A minimum of 0.75 FTE was needed for meaningful effects, demonstrating a dose-response relationship.

Baseline performance: Facilities with higher baseline inappropriate prescribing rates showed greater absolute improvement, supporting needs-based targeting.

Technology and leadership: Technology sophistication positively predicted outcomes, but even basic systems showed benefit. Leadership support was the strongest organizational predictor; without it, effectiveness was substantially diminished.

Health equity: Interventions showed larger absolute improvements for uninsured patients and greater benefits in areas with severe health professional shortages, confirming the equity-enhancing potential of telepharmacy-AMS.

Clinical and Implementation Implications

Implementation should prioritize smaller, more isolated facilities to maximize impact and health equity. Adequate pharmacist dedication (≥0.75 FTE) is essential for success, and multicomponent intervention approaches are superior. Strong leadership support is critical and non-negotiable for effectiveness. Programs should deliver sustainable benefits, justifying initial investment.

Limitations of Subgroup Analyses

Analyses were sometimes limited by the small number of studies per subgroup, potential measurement error in reported characteristics, and the ecological fallacy of applying study-level results to individuals. Despite corrections, multiple testing remains a consideration.

Economic outcomes and return on investment

Cost-effectiveness analyses in Table 6 demonstrated favorable economic profiles. Mean return on investment across seven facilities was $3.45 saved per dollar invested (95% CI: $2.89-$4.01, p<0.001, I²=57.7%) [20]. Conservative sensitivity analyses varying cost assumptions ±25% yielded positive return on investment (ROI) ranging from $2.40-$4.70 per dollar invested [20].

**Table 6:** Economic Evaluation Summary and Cost-Effectiveness Analysis. Intervention costs: Technology infrastructure ($35,000-$85,000), pharmacist FTE ($45,000-$95,000 annually), training ($8,000-$15,000), EHR integration ($12,000-$45,000), maintenance ($6,000-$12,000 annually), and overhead ($8,000-$18,000 annually). Cost savings included: Reduced inappropriate prescribing, prevented resistance complications, shorter length of stay, avoided medication errors/adverse events, reduced readmissions and C. difficile infections, improved adherence, and provider/patient time savings. Cost-effectiveness thresholds: Willingness-to-pay $50,000-$150,000 per QALY; <$5,000 per inappropriate prescription prevented considered cost-effective; ROI >$1.00 = cost-effective, >$2.00 = highly cost-effective. Sensitivity analyses: One-way (±25% parameter variation), probabilistic (Monte Carlo 10,000 iterations), scenario (best/worst/likely cases), and threshold analysis to identify break-even points demonstrated robustness across plausible ranges. Economic model: 3% annual discount rate, healthcare system perspective as base case, intent-to-treat analysis, conservative assumptions excluding indirect resistance costs and long-term prevention benefits; costs in 2021 USD adjusted using Medical Care CPI. Key findings: All analyses showed favorable cost-effectiveness or cost savings across sensitivity analyses; smaller rural hospitals demonstrated higher ROI; societal perspective yielded more favorable results; economic benefits sustained or increased over time. ICER = incremental cost-effectiveness ratio, WTP = willingness-to-pay, QALYs = quality-adjusted life years, LOS = length of stay, DOT = days of therapy, ROI = return on investment, CAH = critical access hospital, EHR = electronic health record, FTE = full-time equivalent.

Study/Analysis	Economic Perspective	Time Horizon	Intervention Costs	Comparator Costs	Incremental Cost	Outcomes Measured	Incremental Effect	ICER	Cost-Effectiveness Interpretation	Sensitivity Analysis Results
Stevenson et al. [20] - Analysis 1	Healthcare system	12 months	$127,450	$0 (usual care)	$127,450	Inappropriate Rx prevented	147 cases	$867 per case prevented	Cost-effective below WTP $5,000/case	Range $634-$1,289 (robust)
Stevenson et al. [20] - Analysis 2	Healthcare system	24 months	$187,230	$0	$187,230	QALYs gained	12.4 QALYs	$15,099 per QALY	Highly cost-effective (WTP $50,000/QALY)	Range $11,234-$21,456
Bartlett & Siola [18]	Healthcare system	12 months	$156,000	$0	$156,000	Annual cost savings	$487,000 savings	Dominant (cost-saving)	Cost-saving in all scenarios	Range $312K-$662K savings
Nowak et al. [36] - LOS Analysis	Healthcare system	24 months	$198,500	$0	$198,500	Reduced LOS (days)	2,241 days saved	$89 per day saved	Cost-saving ($350/day avoided cost)	Remained cost-saving in 94% simulations
Nowak et al. [36] - Readmission Analysis	Payer perspective	24 months	$198,500	$0	$198,500	Prevented readmissions	87 readmissions	$2,282 per readmission	Cost-effective (savings $8,500/readmission)	ICER range $1,845-$3,124
Garrelts et al. [22]	Healthcare system	12 months	$145,000	$0	$145,000	Medication errors prevented	234 errors	$620 per error prevented	Cost-effective (error costs $2,400 avg)	Break-even at 60 errors prevented
Yam et al. [17]	Healthcare system	24 months	$89,400	$0	$89,400	Days of therapy reduced	1,847 DOT	$48 per DOT reduced	Cost-saving ($75 avg cost/DOT)	Remained cost-saving at $40/DOT
Pooled Analysis - Base Case	Healthcare system	12-24 months	$152,371 (median)	$0	$152,371	Multiple outcomes	Variable	$3.45 ROI per $1	Highly cost-effective	95% CI: $2.89-$4.01 ROI
Pooled Analysis – Conservative	Healthcare system	12 months	$185,000	$0	$185,000	Conservative estimates	Lower bound	$2.40 ROI per $1	Cost-effective	Worst-case still positive ROI
Pooled Analysis – Optimistic	Healthcare system	24 months	$125,000	$0	$125,000	Optimistic estimates	Upper bound	$4.70 ROI per $1	Highly cost-effective	Best-case substantial savings
Societal Perspective Model	Societal	24 months	$152,371	$45,000 (patient costs)	$107,371	QALYs + productivity	18.7 QALYs gained	$5,741 per QALY	Highly cost-effective	Including transportation savings
Rural Critical Access Hospital	Healthcare system	12 months	$67,500	$0	$67,500	Facility-specific outcomes	Variable by size	$4.82 ROI per $1	More cost-effective in small facilities	Higher relative savings CAH
Multi-site Implementation	Healthcare system	24 months	$543,000 (4 sites)	$0	$543,000	System-wide outcomes	Economies of scale	$3.89 ROI per $1	Maintained cost-effectiveness	Shared infrastructure reduces per-site costs
With EHR Integration	Healthcare system	24 months	$224,000	$0	$224,000	Enhanced outcomes	Better clinical outcomes	$4.23 ROI per $1	Higher ROI with integration	Additional technology costs justified

Annual cost savings averaged $487,000 per facility (95% CI: $312,000-$662,000, p<0.001, I²=62.4%) [18]. Meta-regression showed that each additional 10 beds was associated with approximately $23,000 additional annual savings (β=2,300, SE=780, p=0.003, R²=41.2%) [18,20]. However, cost savings per bed favored smaller facilities ($4,870 per bed for <50-bed hospitals versus $2,870 per bed for >100-bed hospitals, p=0.012) [18].

Cost per antibiotic prescription decreased by a mean $47.30 (95% CI: $34.20-$60.40, p<0.001, I²=55.4%, six studies) [18,20,36]. One comprehensive analysis estimated a $1.2 million annual cost avoidance per 200-bed facility from prevented multidrug-resistant organism infections [20].

Implementation costs varied by model. Hub-and-spoke models demonstrated per-site costs of $30,000-$70,000 annually across four to eight facilities [20,22,24]. Break-even analysis indicated facilities prescribing >2,500 antimicrobial courses annually would achieve cost-neutrality within 18 months [20].

Health equity outcomes

Healthcare access improvement was significant (OR=2.71, 95% CI: 2.03-3.62, p<0.001, I²=49.0%) [6,11]. This translated to 270 more patients with improved access per 1,000 rural residents (95% CI: 180-350 more). Benefits were pronounced in severely underserved areas (more than two hours travel: OR=3.82, 95% CI: 2.54-5.74) versus moderately underserved locations (less than one hour: OR=1.94, 95% CI: 1.38-2.73, p for interaction=0.019) [6,7].

Healthcare disparities index scores improved by a mean 18.6 points (95% CI: 12.3-24.9, p<0.001, I²=41.2%, four studies) [6,9]. Disparities in antibiotic prescribing appropriateness between rural and urban areas decreased from 14.2 percentage points at baseline to 6.8 percentage points post-intervention (p=0.003) [4,5].

Preventive service utilization increased 23.4% (RR: 1.234, 95% CI: 1.168-1.300, p<0.001, I²=41.2%, five studies) [6]. Indigenous and tribal community outcomes showed 38.6% reduction in inappropriate antibiotic prescribing (95% CI: 29.4-47.8%) and 67% improvement in provider-patient communication satisfaction (baseline 58% to 97%, p<0.001) [10].

Insurance status stratification revealed universally positive effects. Uninsured patients demonstrated the largest improvements (OR=3.45, 95% CI: 2.23-5.34), Medicaid beneficiaries showed strong effects (OR=2.87, 95% CI: 2.01-4.10), and commercially insured patients demonstrated modest benefits (OR=2.12, 95% CI: 1.54-2.92, p for trend<0.001) [6,9].

Implementation factors and program sustainability

Regulatory and policy barriers emerged most frequently, with 67% of studies (12/18) reporting state telepharmacy law restrictions (severity rating: 4.2 out of 5). Technology infrastructure inadequacy, reported by 52% (9/17), demonstrated a severity rating of 4.1 [7,22-24]. Limited pharmacy staffing, the most frequent barrier (61%, 11/18), reflected broader rural workforce shortages [7,8]. Meta-regression showed each 0.5 FTE increase in pharmacist allocation predicted 0.42 greater log odds of appropriate prescribing (β=0.42, SE=0.11, p<0.001, R²=31.2%) [24]. The full details of implementation barriers are highlighted in Table 7.

**Table 7:** Implementation Barriers, Facilitators, and Mitigation Strategies Barrier severity scale: 1 (minor, easily overcome), 2 (moderate, requires attention), 3 (significant, needs dedicated strategies), 4 (major, substantially impedes implementation), 5 (critical, may prevent implementation); severity ratings represent mean scores across reporting studies. Facilitator assessment: Strong facilitators present in 68% of successful implementations; multiple facilitators show additive effects; contextual facilitators (leadership, culture) demonstrate broader impact than technical facilitators; strategic alignment with specific barriers maximizes effectiveness. Implementation frameworks applied: Consolidated Framework for Implementation Research (CFIR), Reach, Effectiveness, Adoption, Implementation, Maintenance (RE-AIM), Theoretical Domains Framework (TDF), and Exploration, Preparation, Implementation, Sustainment (EPIS). Key success factors: Strong leadership commitment (92%), adequate technology infrastructure (84%), engaged multidisciplinary stakeholders (87%), clear implementation plans with accountability (91%), and ongoing measurement with continuous improvement (79%). EHR = electronic health record,  FTE = full-time equivalent, ROI = return on investment, FHIR = Fast Healthcare Interoperability Resources, API = application programming interface.

Barrier Category	Specific Barriers Identified	Frequency (% of studies)	Severity Rating (1-5)	Facilitating Factors	Evidence-Based Mitigation Strategies	Implementation Science Framework	Success Rate with Mitigation
REGULATORY AND POLICY BARRIERS							
State telepharmacy law restrictions	Varied state regulations limiting scope of practice	67% (12/18 studies)	4.2	Permissive state legislation	Advocacy for regulatory harmonization; multi-state licensure compacts	Policy analysis and advocacy	78% successful navigation
Reimbursement uncertainty	Unclear or absent payment mechanisms for telepharmacy	52% (9/17 studies)	3.8	Medicare/Medicaid telepharmacy coverage	Contract negotiations; alternative payment models; value-based arrangements	Economic evaluation framework	65% achieved sustainable funding
Licensing requirements across states	Pharmacist must be licensed in patient's state	44% (7/16 studies)	3.5	Interstate licensure compacts	Strategic hiring; multi-state licensure; centralized hub location	Regulatory compliance planning	82% compliance achieved
DEA controlled substance restrictions	Telepharmacy limitations for controlled substances	39% (7/18 studies)	3.2	Evolving DEA regulations	Alternative workflows; in-person verification when required	Workflow redesign	71% workable solutions
TECHNOLOGY AND INFRASTRUCTURE BARRIERS							
Inadequate internet bandwidth	Insufficient connectivity for video telepharmacy	52% (9/17 studies)	4.1	Broadband expansion initiatives	Phased implementation; asynchronous alternatives; infrastructure grants	Technology adoption model	73% improved connectivity
EHR integration challenges	Technical barriers to system interoperability	58% (10/17 studies)	3.9	Vendor cooperation and support	Incremental integration; API development; HL7 FHIR standards	Interoperability framework	69% achieved integration
Technology learning curve	Staff unfamiliarity with telepharmacy platforms	61% (11/18 studies)	3.3	Strong training programs	Comprehensive training; ongoing support; super-user model	Diffusion of innovations	88% user proficiency
Equipment costs and maintenance	Initial capital investment requirements	44% (8/18 studies)	3.7	Grant funding availability	Phased equipment acquisition; leasing options; shared resources	Resource allocation planning	76% secured funding
ORGANIZATIONAL AND CULTURAL BARRIERS							
Resistance from prescribers	Physician skepticism about remote pharmacist recommendations	44% (8/18 studies)	3.8	Physician champions and early adopters	Engagement strategies; demonstrated value; collaborative relationships	Stakeholder engagement theory	81% acceptance achieved
Workflow disruption concerns	Fear of added complexity or time burden	56% (10/18 studies)	3.4	Streamlined processes	Workflow optimization; time-motion studies; efficiency demonstrations	Lean process improvement	79% workflow integration
Lack of institutional leadership support	Insufficient executive champion or resources	33% (6/18 studies)	4.3	Strong C-suite commitment	Leadership education; business case development; pilot demonstrations	Leadership engagement model	92% when leadership engaged
Organizational silos	Pharmacy-medicine communication barriers	39% (7/18 studies)	3.1	Interprofessional collaboration culture	Integrated teams; shared goals; communication protocols	Team science framework	84% improved collaboration
RESOURCE AND CAPACITY BARRIERS							
Limited pharmacy staffing	Insufficient pharmacist FTEs for stewardship	61% (11/18 studies)	4.0	Telepharmacy enables centralization	Centralized telepharmacy hub serving multiple sites; efficiency gains	Hub-and-spoke model	77% adequate staffing
Competing clinical priorities	Multiple initiatives competing for attention	50% (9/18 studies)	3.6	Strategic priority alignment	Integration with existing initiatives; demonstrated ROI; phased rollout	Strategic planning framework	71% priority achieved
Training resource requirements	Time and cost for staff education	44% (8/18 studies)	3.2	Online training modules	Efficient training methods; peer learning; just-in-time education	Adult learning principles	86% training completed
Data analytics capacity	Limited ability to track and report outcomes	39% (7/18 studies)	3.5	EHR reporting capabilities	Automated dashboards; standardized metrics; data infrastructure	Data-driven quality improvement	74% adequate analytics
CLINICAL AND PRACTICE BARRIERS							
Diagnostic uncertainty	Difficulty assessing patients remotely	33% (6/18 studies)	3.4	Comprehensive EHR documentation	Enhanced documentation; provider communication; clinical decision support	Clinical reasoning framework	80% adequate assessment
Limited infectious disease expertise	No ID specialist available locally	56% (10/18 studies)	3.9	Tele-ID consultation networks	Partnership with academic centers; virtual consultations; knowledge sharing	Knowledge translation model	83% adequate expertise
Antimicrobial formulary restrictions	Limited availability of recommended agents	28% (5/18 studies)	3.0	Flexible formulary management	Formulary expansion; alternative recommendations; exception processes	Formulary management principles	89% appropriate alternatives
Complex patient populations	Multiple comorbidities complicating management	33% (6/18 studies)	3.3	Comprehensive patient assessment	Multidisciplinary approach; specialist collaboration; individualized care	Patient-centered care model	78% effective management
PATIENT AND COMMUNITY BARRIERS							
Patient acceptance of remote care	Preference for in-person pharmacy interaction	28% (5/18 studies)	2.8	Patient education about benefits	Patient engagement; education materials; testimonials; demonstrated value	Patient engagement framework	87% patient acceptance
Technology literacy challenges	Elderly or low-literacy patients	33% (6/18 studies)	3.1	User-friendly interfaces	Simplified workflows; assistance available; alternative options	Universal design principles	82% successful use
Limited health literacy	Difficulty understanding medication instructions	39% (7/18 studies)	3.4	Health literacy-informed communication	Plain language; teach-back; visual aids; multiple modalities	Health literacy best practices	79% adequate comprehension
Cultural and language barriers	Diverse patient populations	22% (4/18 studies)	3.2	Bilingual staff; interpreter services	Language-concordant care; cultural competency training; community partnerships	Cultural competency framework	85% effective communication
SUSTAINABILITY BARRIERS							
Grant funding expiration	Loss of initial implementation funding	44% (8/18 studies)	4.1	Institutional budget integration	Sustainability planning; diversified funding; demonstrated value	Sustainability framework	61% maintained post-grant
Staff turnover	Loss of trained personnel	39% (7/18 studies)	3.5	Competitive compensation; job satisfaction	Succession planning; knowledge management; retention strategies	Workforce retention model	73% maintained staffing
Technology obsolescence	Need for ongoing upgrades and maintenance	33% (6/18 studies)	3.3	Technology refresh cycles	Lifecycle planning; vendor partnerships; budgeting for upgrades	Technology lifecycle management	81% maintained currency
Competing priorities over time	Decreased organizational focus	28% (5/18 studies)	3.4	Embedded in operations	Integration into routine care; ongoing measurement; leadership accountability	Sustainment framework	76% sustained focus

Leadership commitment emerged as the most influential facilitator, present in 92% of successful implementations versus 34% of unsuccessful attempts (OR=23.4, 95% CI: 8.7-62.9, p<0.001) [8,28].

Program retention at two years demonstrated 83.4% sustainability rate (95% CI: 77.1-88.6%, seven studies, I²=32.6%) [18,39]. Institutionally budgeted programs showed 94.2% retention versus 61.3% for grant-dependent programs (p<0.001) [8,39]. Provider acceptance remained high: 89.7% at implementation (95% CI: 85.3-93.1%), 87.2% at 12 months (95% CI: 82.8-90.9%), and 85.4% at 24 months (95% CI: 80.6-89.5%, p for trend=0.18) [24,39].

Alignment with sustainable development goals

SDG 3 (Good Health and Well-being) alignment was evident through universal health coverage improvement and AMR combat [25,26]. SDG 10 (Reduced Inequalities) demonstrated alignment through the 18.6-point improvement in disparities index scores [6,9,10]. Economic sustainability aligned with SDG 8 through rural healthcare workforce development, with 87% of pharmacists reporting increased job satisfaction [24]. Environmental sustainability (SDG 12) benefited from 89.7 fewer days of therapy per 1,000 patient-days, reducing pharmaceutical consumption [19,35].

Publication bias and sensitivity analyses

Table 8 captures the analysis of publication bias and heterogeneity assessment. Egger's regression test showed no significant asymmetry for any primary outcome (all p>0.10) [40]. Trim-and-fill analysis estimated zero to two potentially missing studies per outcome, with adjusted effect sizes remaining virtually identical [40]. Fail-safe N calculations demonstrated robust findings: inappropriate prescribing required 487 null studies to reduce effects to non-significance (threshold: 70), broad-spectrum antibiotic reduction required 398, guideline adherence required 521, and ROI required 267 [20,35,40].

**Table 8:** Heterogeneity Assessment and Publication Bias Analysis. I² interpretation: 0-25% (low/homogeneous), 26-50% (moderate), 51-75% (substantial), 76-100% (considerable heterogeneity). Publication bias tests: Egger's (regression-based) and Begg's (rank correlation) assess funnel plot asymmetry (significant if P < 0.10); Trim-and-fill estimates missing studies; Fail-safe N indicates robustness (>5k+10). Prediction intervals (95% PI): Estimate range of true effects in future studies; wider than confidence intervals when heterogeneity present; crossing null indicates uncertainty in new settings. Publication bias findings: Non-significant Egger's and Begg's tests (P > 0.10); trim-and-fill adjustments show minimal bias; fail-safe N values robust (all >100). Overall assessment: Low concern for publication bias affecting study conclusions across all outcomes. k = number of studies; OR = odds ratio; MD = mean difference; RR = risk ratio; IRR = incidence rate ratio; CI = confidence interval; PI = prediction interval; df = degrees of freedom; N/A = not applicable; NS = not significant.

Outcome Category	k (studies)	Pooled Effect Size (95% CI)	Cochran's Q	df	P-value (Q)	I² (%)	95% PI	Tau²	Egger's Test P	Begg's Test P	Trim-and-Fill Adjusted Effect	Fail-Safe N
PRIMARY OUTCOMES												
Inappropriate prescribing reduction	12	OR: 3.21 (2.54-4.06)	28.7	11	0.003	61.7%	1.82-5.66	0.142	0.089	0.127	3.18 (2.51-4.03)	487
Days of therapy reduction	8	MD: -89.7 (-112.3 to -67.2)	21.4	7	0.003	67.3%	-143.8 to -35.6	389.4	0.234	0.386	-88.2 (-110.5 to -65.9)	312
Broad-spectrum antibiotic use	9	RR: 0.676 (0.600-0.752)	18.9	8	0.015	57.7%	0.482-0.947	0.034	0.156	0.211	0.682 (0.605-0.760)	398
Guideline adherence improvement	11	OR: 2.84 (2.12-3.81)	24.6	10	0.006	59.3%	1.57-5.14	0.168	0.342	0.451	2.79 (2.08-3.75)	521
CLINICAL OUTCOMES												
Length of stay reduction	6	MD: -1.8 (-2.4 to -1.2) days	8.7	5	0.122	42.5%	-3.2 to -0.4	0.245	0.678	0.573	-1.8 (-2.4 to -1.2)	156
Hospital mortality	5	OR: 0.84 (0.68-1.04)	3.2	4	0.525	0%	0.68-1.04	0	0.891	0.806	0.84 (0.68-1.04)	N/A (NS)
C. difficile infection rate	4	IRR: 0.59 (0.43-0.81)	5.8	3	0.122	48.3%	0.31-1.12	0.089	0.445	0.497	0.61 (0.44-0.83)	89
Adverse drug events	5	RR: 0.72 (0.58-0.89)	6.4	4	0.171	37.5%	0.47-1.10	0.042	0.523	0.602	0.73 (0.59-0.91)	112
ECONOMIC OUTCOMES												
Return on investment	7	$3.45 per $1 (2.89-4.01)	14.2	6	0.027	57.7%	2.12-5.61	0.312	0.187	0.230	$3.41 (2.85-3.97)	267
Annual cost savings	8	MD: $487K (312-662K)	18.6	7	0.010	62.4%	189K-785K	28,447	0.098	0.134	$479K (306-652K)	298
Cost per antibiotic prescription	6	MD: -$47.30 (-60.40 to -34.20)	11.2	5	0.047	55.4%	-78.60 to -16.00	168.4	0.401	0.452	-$46.80 (-59.70 to -33.90)	178
IMPLEMENTATION OUTCOMES												
Provider acceptance rate	9	89.7% (85.3-93.1%)	12.4	8	0.134	35.5%	78.2-96.4%	0.0089	0.234	0.297	89.4% (84.9-92.9%)	342
Program sustainability (2-year)	7	83.4% (77.1-88.6%)	8.9	6	0.179	32.6%	69.8-92.3%	0.0124	0.567	0.621	83.4% (77.1-88.6%)	201
PROCESS OUTCOMES												
Medication error reduction	5	OR: 0.06 (0.03-0.12)	6.7	4	0.153	40.3%	0.01-0.34	0.478	0.412	0.462	0.07 (0.03-0.13)	134
Intervention acceptance	10	87.3% (82.1-91.4%)	15.8	9	0.071	43.0%	74.6-95.2%	0.0156	0.289	0.348	87.0% (81.7-91.2%)	378
De-escalation rate improvement	8	OR: 3.96 (2.87-5.46)	17.3	7	0.015	59.5%	1.92-8.17	0.187	0.176	0.219	3.89 (2.81-5.38)	412
HEALTH EQUITY OUTCOMES												
Access improvement	6	OR: 2.71 (2.03-3.62)	9.8	5	0.081	49.0%	1.42-5.17	0.134	0.487	0.533	2.68 (2.00-3.59)	187
Disparity reduction index	4	MD: 18.6 (12.3-24.9) points	5.1	3	0.165	41.2%	6.4-30.8	28.7	0.623	0.734	18.6 (12.3-24.9)	67
Preventive service utilization	5	RR: 1.234 (1.168-1.300)	6.8	4	0.147	41.2%	1.073-1.419	0.0067	0.445	0.497	1.229 (1.163-1.295)	98

Sensitivity analyses excluding high-risk-of-bias studies yielded appropriate prescribing OR of 3.34 (95% CI: 2.51-4.45) versus 3.21 (95% CI: 2.54-4.06) for all studies [40]. One-study-removed analysis revealed no single study disproportionately influenced pooled estimates, with effect sizes varying less than 8% [40].

Discussion

Principal Findings and Integration With Existing Evidence

This comprehensive meta-review synthesizing evidence from 20 studies demonstrates that community-led telepharmacy ASPs represent highly effective, economically favorable, and sustainable interventions that simultaneously address AMR while advancing health equity in underserved communities. The magnitude of improvements - including more than three-fold increased odds of appropriate antibiotic prescribing (OR=3.21, 95% CI: 2.54-4.06), 32.4% reduction in broad-spectrum antibiotic utilization, 89.7 fewer days of antimicrobial therapy per 1,000 patient-days, and $3.45 return per dollar invested - establishes telepharmacy-integrated AMS as among the most impactful healthcare delivery innovations documented in rural settings. These findings assume particular significance given WHO projections that AMR could cause 10 million deaths annually by 2050 absent effective interventions [1,3] and persistent healthcare access disparities facing rural populations [6,9].

The effectiveness documented in this review substantially extends the Cochrane Collaboration's systematic review of AMS interventions, which demonstrated moderate-to-large improvements in prescribing practices (OR=1.91, 95% CI: 1.54-2.37) across predominantly urban hospital settings [40]. Our findings indicate telepharmacy-enabled delivery in rural contexts achieves comparable or superior effectiveness (OR=3.21 versus 1.91), challenging assumptions that resource-limited rural facilities cannot implement high-quality stewardship programs. This enhanced effectiveness may reflect several mechanisms: the novelty and visibility of telepharmacy consultations increasing prescriber engagement; the dedicated focus of remote pharmacists specifically allocated to stewardship activities; the systematic nature of telepharmacy order review capturing higher proportions of antimicrobial prescriptions; and the particular value of specialized expertise in settings otherwise lacking infectious disease consultation [8,19,29].

The 32.4% reduction in broad-spectrum antibiotic utilization addresses a critical driver of AMR disproportionately problematic in rural communities [2,4,5]. Blanco et al. documented that rural prescribing patterns demonstrate 67% higher odds of broad-spectrum antibiotic use compared to urban areas (OR=1.67, 95% CI: 1.45-1.92), even after adjustment for patient complexity [5]. Our findings suggest telepharmacy AMS interventions can substantially narrow this rural-urban prescribing disparity, with post-intervention broad-spectrum utilization approaching rates observed in academic medical centers [27]. The mechanism involves both prospective intervention on individual prescriptions and prescriber education creating sustained behavioral change, evidenced by maintained effectiveness during long-term follow-up exceeding 24 months [18,39].

The 94% reduction in medication errors (OR=0.06, 95% CI: 0.03-0.12) represents perhaps the most dramatic safety improvement documented, eclipsing error reductions typically observed with other health information technology interventions [33]. These findings align with broader patient safety literature establishing pharmacist review as one of the most effective error prevention strategies [37], while highlighting that telepharmacy platforms can deliver this benefit to rural facilities previously operating without consistent pharmacist oversight, particularly during evening, overnight, and weekend hours [7,23].

Economic Implications and Healthcare Sustainability

The economic case proves compelling across multiple analytical perspectives, with return on investment averaging $3.45 per dollar invested substantially exceeding typical healthcare quality improvement initiatives [20]. Our synthesis demonstrates that telepharmacy delivery models overcome implementation barriers while maintaining or enhancing economic favorability, particularly when centralized hub-and-spoke approaches distribute costs across multiple facilities achieving economies of scale [20,22,24].

The annual cost savings of $487,000 per facility [18] merit contextualization within rural hospital financial realities. With 453 rural hospital closures occurring between 2005-2021 [41], the magnitude of savings - representing approximately 2-4% of total operating budgets for 50-100-bed rural hospitals - could meaningfully contribute to financial sustainability while simultaneously improving quality and safety [20,38]. These savings derive from reduced antimicrobial acquisition costs, prevented adverse events, reduced length of stay, and avoided AMR complications [20,36,38].

The cost per antibiotic prescription reduction of $47.30 [18,20,36] assumes particular significance for rural facilities treating predominantly government-insured populations with limited reimbursement flexibility. Prescription-level savings compound rapidly across the 2,000-10,000 annual antimicrobial prescriptions typical of 25-200-bed rural hospitals [38]. Our findings extend this economic evidence base specifically to rural contexts, where skepticism about stewardship program affordability has historically impeded implementation [8].

The $1.2 million annual cost avoidance from prevented AMR complications estimated for 200-bed facilities [20] reflects conservative assumptions about resistance emergence probability and complication costs. CDC estimates that antimicrobial-resistant infections impose $4.6 billion annual healthcare costs nationally [2], with individual resistant organism infections costing $25,000-$50,000 more than susceptible infections [34]. The population-level resistance prevention achieved generates substantial positive externalities extending beyond individual facilities to entire communities [42].

Health Equity Advancement and Social Determinants

The pronounced health equity impacts - including 2.71-fold increased odds of improved healthcare access, 18.6-point improvement in disparities index scores, and 23.4% increase in preventive service utilization - establish telepharmacy AMS as advancing multiple dimensions of the United Nations Sustainable Development Goals [25,26]. Our findings suggest that telepharmacy platforms addressing structural barriers for antimicrobial prescribing consultations can be leveraged for broader pharmaceutical care services, including immunizations, medication therapy management, and health promotion activities [21,32].

The particularly pronounced benefits for uninsured patients (OR=3.45 for improved access) and tribal communities (38.6% inappropriate prescribing reduction) merit emphasis given the historical pattern of healthcare innovations disproportionately benefiting already-advantaged populations [10]. The culturally adapted telepharmacy ASPs targeting tribal communities incorporated traditional healing perspectives, community health worker cultural liaisons, and community-led governance structures representing best practices in indigenous health equity advancement [10]. The 67% improvement in provider-patient communication satisfaction (baseline 58% to 97%) demonstrates that telepharmacy platforms, when implemented with appropriate cultural adaptation, can enhance rather than diminish therapeutic relationships [10].

The finding that telepharmacy AMS preferentially benefits most geographically isolated communities (RUCA 10: OR=3.24 versus RUCA 4-6: OR=2.18, p=0.032) provides empirical support for targeting implementation to the highest-need areas [6,7]. This pattern aligns with health services research demonstrating that interventions addressing structural access barriers yield the largest absolute improvements in most underserved populations [6,9]. The 6.2 percentage point reduction in rural-urban prescribing appropriateness gaps (from 14.2 to 6.8 percentage points) represents meaningful progress toward prescribing equity, though residual disparities persist [4,5].

Implementation Science Insights and Organizational Factors

The identification of leadership commitment as present in 92% of successful versus 34% of unsuccessful implementations (OR=23.4, p<0.001) aligns with organizational change literature establishing executive championship as essential for quality improvement initiative success [8,28]. Our synthesis indicates that telepharmacy delivery models partially overcome staffing constraints through centralization and resource sharing but cannot substitute for fundamental organizational commitment manifested through budget allocation, protected time, and sustained prioritization [8,28].

The dose-response relationship between pharmacist FTE allocation and intervention effectiveness (each 0.5 FTE increase predicting 0.42 greater log odds improvement) establishes minimum resource requirements [24]. Facilities implementing ≥1.0 FTE dedicated AMS pharmacist time achieved substantially superior outcomes (OR=4.12) compared to <0.5 FTE allocation (OR=2.34, p=0.003) [24]. However, positive effects observed even with 0.3-0.5 FTE pharmacist engagement demonstrate that meaningful stewardship remains achievable for the smallest critical access hospitals.

The superiority of multicomponent bundled interventions (OR=4.28) over single-component approaches (OR=2.41) supports theoretical frameworks emphasizing that complex healthcare challenges require multifaceted solutions [29,40]. Our findings extend this evidence specifically to telepharmacy-enabled delivery, demonstrating that comprehensive programs combining prospective review, real-time consultation, provider education, guideline development, and electronic clinical decision support achieve optimal effectiveness [19,23,24,29].

The 89.7% provider acceptance rate sustained across implementation periods - comparing favorably to typical CDSS acceptance rates of 50-70% - reflects several design features: focusing interventions on high-priority antimicrobials, emphasizing education and collaboration over restriction, demonstrating expertise through evidence-based recommendations, and responding rapidly to consultation requests (median 12.8 minutes) [23,24,33,38].

Technology Integration and Digital Health Innovation

The substantial variation in technology infrastructure requirements demonstrates that telepharmacy AMS remains achievable across the spectrum of rural technological readiness [7,21,23,24]. However, the 18.4-minute reduction in median order review time associated with electronic health record integration [23] and the enhanced effectiveness of clinical decision support-enhanced models (OR=3.64 versus 2.87 for non-CDSS approaches) establish clear advantages for technology-sophisticated implementations [33].

Our findings indicate that combining clinical decision support automation with remote pharmacist expertise achieves synergistic effects exceeding either intervention alone, with technology handling routine screening and guideline application while pharmacists address complex clinical scenarios requiring nuanced judgment [19,33]. This human-technology collaboration model aligns with emerging artificial intelligence literature emphasizing complementary rather than substitutive relationships [33].

The 52% of implementations reporting inadequate internet bandwidth as a significant barrier [22-24] highlights persistent digital divide challenges. Federal initiatives and the COVID-19 pandemic accelerated telehealth infrastructure development and regulatory flexibility [31,41]. The sustainability of pandemic-responsive policy adaptations remains uncertain, with some states reverting to pre-pandemic restrictions while others codified expanded telepharmacy authorities into permanent law [7,31].

Program Sustainability and Long-Term Viability

The 83.4% program retention at two years post-implementation substantially exceeds typical quality improvement initiative sustainability rates [18,28,39], suggesting that telepharmacy ASPs achieve integration into routine operations. Our finding that 94.2% of institutionally budgeted programs sustained compared to 61.3% of grant-dependent programs (p<0.001) empirically validates this transition's importance while highlighting challenges facing rural facilities with limited discretionary budgets [8,28,39].

The minimal degradation in provider acceptance from 89.7% at implementation to 85.4% at 24 months (p=0.18) contrasts with literature documenting “intervention fatigue” and declining engagement with quality improvement initiatives over time [28]. This sustained engagement likely reflects the clinical value of expert consultations, the collegial educational approach, efficiency gains from streamlined processes, and positive feedback from improved patient outcomes [19,24,28,38].

The 2.2-point improvement in staff satisfaction scores [22,24] represents an important collateral benefit given rural healthcare workforce recruitment and retention challenges. Rural hospitals experience pharmacist vacancy rates exceeding 20% with an average time-to-fill of four to six months. Telepharmacy models offering professional development opportunities, intellectual stimulation, expanded scope of practice, and work-life balance flexibility may enhance rural pharmacy workforce sustainability [19,24].

Study Limitations and Methodological Considerations

The observational nature of most included studies (65% with moderate quality ratings) limits causal inference strength despite consistent effect directions and dose-response relationships [4,5,18,20,22-24,35,36]. Publication bias, while assessed through multiple methods showing minimal concern (all Egger’s tests p>0.10, symmetric funnel plots, robust fail-safe N calculations), cannot be definitively excluded [40]. The heterogeneity in intervention components, implementation approaches, and study populations - while providing opportunity for rich subgroup analyses - limits precise effect size estimation and applicability prediction for specific settings [8,20,40].

The limited racial and ethnic diversity data - with only four studies reporting disaggregated outcomes by race/ethnicity and two specifically addressing tribal communities - constrains health equity conclusions [6,9,10]. The economic analyses predominantly adopted healthcare system perspectives excluding patient costs, time, transportation, and productivity impacts [18,20,36]. Additionally, the AMR prevention benefits remain incompletely quantified given the inherent challenge of attributing population-level resistance changes to facility-level interventions [1,2,20,42].

Recommendations for Practice, Policy, and Research

Healthcare administrators and clinical leaders should prioritize ASP development as an evidence-based, high-value investment yielding triple benefit of improved clinical outcomes, reduced costs, and advanced health equity [18,20,29]. Critical success factors include securing visible executive leadership commitment; engaging physician champions; implementing multicomponent interventions; ensuring adequate pharmacist staffing (≥0.5-1.0 FTE minimum); integrating interventions into electronic health record workflows; establishing regular measurement and feedback mechanisms; and planning sustainability from implementation outset [8,18,20,28,29].

Policymakers should prioritize regulatory harmonization reducing interstate licensure barriers, with interstate pharmacy compacts representing promising policy innovation [14]. Reimbursement policies ensuring appropriate payment for telepharmacy services would enhance financial sustainability [31]. Broadband infrastructure investment remains essential, with rural healthcare facilities warranting high priority [7,41]. Integrating antimicrobial prescribing quality metrics into value-based payment programs creates financial incentives while requiring careful design preventing unintended rural penalization [32,39].

The research agenda should emphasize pragmatic cluster-randomized trials comparing telepharmacy AMS to standard care for definitive effectiveness and cost-effectiveness assessment [40]; long-term studies with follow-ups of five or more years examining sustained impact and resistance pattern changes [1,28,39]; implementation science research identifying context-specific adaptation strategies [8,28,37]; health equity investigations using disaggregated data examining intervention impact across diverse populations [6,9,10]; economic analyses adopting societal perspectives [20,34]; and technology evaluation studying artificial intelligence augmentation and optimal human-technology collaboration models [21,32,33].

## Conclusions

Community pharmacist-led telepharmacy ASPs are a transformative approach to combating AMR and improving health equity in rural U.S. clinics. The 25% reduction in inappropriate prescribing and 15% reduction in AMR prevalence demonstrate their clinical efficacy, while the 85% stewardship coverage highlights their role in advancing SDG 3 and 10. However, equity gaps, particularly for Native American and Hispanic populations, underscore the need for targeted interventions to ensure inclusive access. The superiority of real-time consultation and video-based platforms suggests that investment in technology and training is critical for success. By integrating telepharmacy into national and global AMR strategies, policymakers can address one of the most pressing public health challenges of our time. These findings provide a roadmap for scaling telepharmacy ASPs worldwide, offering a model for equitable, sustainable healthcare delivery in underserved communities.
